# Massive QTL analysis identifies pleiotropic genetic determinants for stress resistance, aroma formation, and ethanol, glycerol and isobutanol production in *Saccharomyces cerevisiae*

**DOI:** 10.1186/s13068-021-02059-w

**Published:** 2021-11-02

**Authors:** Ping-Wei Ho, Supinya Piampongsant, Brigida Gallone, Andrea Del Cortona, Pieter-Jan Peeters, Frank Reijbroek, Jules Verbaet, Beatriz Herrera, Jeroen Cortebeeck, Robbe Nolmans, Veerle Saels, Jan Steensels, Daniel F. Jarosz, Kevin J. Verstrepen

**Affiliations:** 1 VIB–KU Leuven Center for Microbiology, Leuven, Belgium; 2 CMPG Laboratory of Genetics and Genomics, Department M2S, KU Leuven, Leuven, Belgium; 3 Leuven Institute for Beer Research, Leuven, Belgium; 4 Department of Chemical and Systems Biology, Stanford University School of Medicine, Stanford, CA 94305 USA; 5 Department of Developmental Biology, Stanford University School of Medicine, Stanford, CA 94305 USA; 6 Labo VIB-CMPG, Bio-Incubator, Gaston Geenslaan 1, 3001 Leuven, Heverlee Belgium

## Abstract

**Background:**

The brewer’s yeast *Saccharomyces cerevisiae* is exploited in several industrial processes, ranging from food and beverage fermentation to the production of biofuels, pharmaceuticals and complex chemicals. The large genetic and phenotypic diversity within this species offers a formidable natural resource to obtain superior strains, hybrids, and variants. However, most industrially relevant traits in *S*. *cerevisiae* strains are controlled by multiple genetic loci. Over the past years, several studies have identified some of these QTLs. However, because these studies only focus on a limited set of traits and often use different techniques and starting strains, a global view of industrially relevant QTLs is still missing.

**Results:**

Here, we combined the power of 1125 fully sequenced inbred segregants with high-throughput phenotyping methods to identify as many as 678 QTLs across 18 different traits relevant to industrial fermentation processes, including production of ethanol, glycerol, isobutanol, acetic acid, sulfur dioxide, flavor-active esters, as well as resistance to ethanol, acetic acid, sulfite and high osmolarity. We identified and confirmed several variants that are associated with multiple different traits, indicating that many QTLs are pleiotropic. Moreover, we show that both rare and common variants, as well as variants located in coding and non-coding regions all contribute to the phenotypic variation.

**Conclusions:**

Our findings represent an important step in our understanding of the genetic underpinnings of industrially relevant yeast traits and open new routes to study complex genetics and genetic interactions as well as to engineer novel, superior industrial yeasts. Moreover, the major role of rare variants suggests that there is a plethora of different combinations of mutations that can be explored in genome editing.

**Supplementary Information:**

The online version contains supplementary material available at 10.1186/s13068-021-02059-w.

## Background

The brewer’s yeast *Saccharomyces cerevisiae* plays a key role in the production of fermented foods, beverages, biofuels and pharmaceuticals. Despite *S. cerevisiae* being generally suited for industrial applications, there is an enormous genetic and phenotypic diversity among different *S. cerevisiae* strains, with strains typically excelling in some areas, but scoring worse for other important phenotypes [1]. Moreover, the strains currently used for industrial production only represent a small fraction of the natural diversity and do not always perform optimally [1–6]. Hence, one obvious yet effective path towards superior industrial yeasts with improved fermentation properties is through the exploration and exploitation of the natural yeast biodiversity [7, 8]. Aside from selecting natural strains that boast a desirable combination of phenotypes, it is also possible to cross multiple strains and select hybrids that combine and improve upon specific traits [7–9]. However, although this approach has proven extremely powerful, it also suffers from important shortcomings. Firstly, natural yeast strains rarely combine all desired traits. Secondly, while hybrids allow combining useful traits, the very process of shuffling genomes is relatively slow and labor-intensive, and inevitably also leads to loss of some positive properties.

An alternative route to exploit the natural diversity of yeasts is to employ genetic engineering to combine specific mutations and alleles that contribute to desirable phenotypes. The use of various CRISPR-based techniques has now made it relatively easy to introduce a series of desirable allele combinations into an existing yeast, to further improve one or several phenotypes while not affecting the rest of the genome and thus maximizing the conservation of other traits [10–12].

Despite the promise of genetic modification and genome editing to obtain superior industrial microbes, identification of the genetic determinants (gene alleles and other genetic variations) that are responsible for desired traits remains a major challenge. Many industrially relevant phenotypes are quantitative, involving multiple genetic loci (called quantitative trait loci, QTLs) that can sometimes show complex genetic interactions [13]. One frequently used approach to dissect QTLs is experimental evolution and re-sequencing (E&R). By monitoring the genetic changes between isogenic populations as they adapt to specific selective pressure across multiple generations, the causative loci underlying the selected trait may be identified [14–16]. Using this approach, studies have for example pinpointed mutations that contribute to ethanol tolerance [17], glycerol utilization [18], and heat resistance [19]. However, although E&R can serve as a powerful tool, it often leads to physiological trade-offs between a selected trait and other aspects of yeast. Additionally, an E&R approach can only be exploited for evolutionarily selectable phenotypes, such as improvements in growth rate, tolerance, or substrate utilization. However, if the desired phenotype is not inherently selectable or counter-productive for cell’s fitness (e.g., overproduction of specific metabolites), it prevents straightforward selection through evolution.

Another approach used to link a complex trait to its genetic underpinning is quantitative trait loci (QTL) mapping [20–23]. QTL mapping takes advantage of *S*. *cerevisiae*’s meiotic recombination efficiency to determine the extent of co-segregation between loci with known positions (genetic variations) and the genetic determinants of the phenotype of interest, whose positions are unknown [24, 25]. This method has been successfully applied to reveal the molecular basis of several quantitative traits such as heat tolerance [26], acetic acid tolerance [27, 28], ethanol tolerance [29, 30] or wine aroma [31].

Despite the ever-increasing list of identified QTLs in yeast and other organisms, QTL mapping generally suffers from low throughput due to the large body of work required to map and confirm one locus for one specific property [32, 33]. Additionally, different studies mostly focus on one phenotype at a time. Hence, it is mostly unclear whether the same QTL might influence multiple phenotypes (positively or negatively). Moreover, because most studies do not map identified QTLs onto the *S*. *cerevisiae* phylogeny, the evolutionary history and distribution of QTLs across the *S*. *cerevisiae* population is also still largely unknown.

Here, we employ the approach first described by She & Jarosz in 2018 that enables systematic identification of QTLs across multiple traits. This method takes advantage of the decreased sequencing cost and the possibilities of high-throughput phenotyping for yeast to map multiple traits in one large-scale mapping effort. Specifically, the approach exploits a large-scale inbreeding crossing scheme and in silico fine-mapping to identify causative variants at single-nucleotide resolution, while maintaining high statistical power. Using this setup, causal variants have been pinpointed across a wide array of ecologically relevant traits, including drug resistance, carbon source utilization, and chemical stressors [34–36]. Here, we combined the power of the 1125 previously sequenced inbred segregants with large-scale phenotyping methods to pinpoint 678 QTLs that together determine 18 different yeast traits relevant to industrial fermentations and biotechnological processes. From this set, we identified several key variants that influence the production of fermentative metabolites, including ethanol, glycerol, isobutanol, acetic acid, and sulfur dioxide, as well as resistance to ethanol, acetic acid, sulfite, and high osmolarity and salinity. The large number of QTLs identified reflects the genetic complexity of the traits we examined. Thanks to the large segregant population, we were able to resolve 254 QTLs to single-nucleotide level (QTN) and an additional 58 to single gene (size of about 1 kb). Interestingly, we show that pleiotropy plays a major role in phenotypic diversity of industrially relevant yeast characteristics. For example, a (previously introduced) auxotrophic marker in one of the parental strains affected 12 traits, while a specific rearrangement in the subtelomeric region of chromosome VII affected at least 5 phenotypes. Lastly, we show that several of the causative mutations exhibit similar phenotypic effects when introduced to other, genetically distinct industrial strains. This not only confirms the accuracy of our approach, but also opens new routes to study complex genetics and genetic interactions as well as to engineer novel, superior industrial yeasts.

## Results

The main aim of this study was to obtain a comprehensive view of the different QTLs that contribute to industrially relevant properties in *S. cerevisiae*. To this end, 1125 F_6_ segregants were obtained from a cross of two phenotypically divergent *S. cerevisiae* strains, RM11-1a, a natural vineyard isolate, and YJM975α, originally isolated from an immunocompromised patient in Italy [34]. We reasoned that this combination of one industrial, and one non-industrial strain would maximize our chances of identifying QTLs that are specific for strains adapted to industrial conditions, while possibly also revealing to what extent non-industrial strains also harbor other industrially relevant alleles. For each of the 1125 segregants and the two parental yeasts, we set up small-scale fermentation reactions in medium and conditions mimicking industrial beer production. For each of these 1127 fermentations, we measured 18 different industrially relevant parameters, including the production of primary and secondary metabolites. In addition, we screened all 1125 segregants for their resistance to various industrially relevant stress factors and consumption of maltose. This large set of phenotypic data was subsequently combined with the available genome sequences of each of the segregants and analyzed using the pipeline developed by She and Jarosz (2018) to identify QTLs underlying the phenotypes. Finally, some of the QTLs were experimentally confirmed to verify the mapping and explore the possibility of using the data to engineer superior industrial yeasts.

### Identification of *LEU2* as a major pleiotropic QTL

**Fig. 1:**
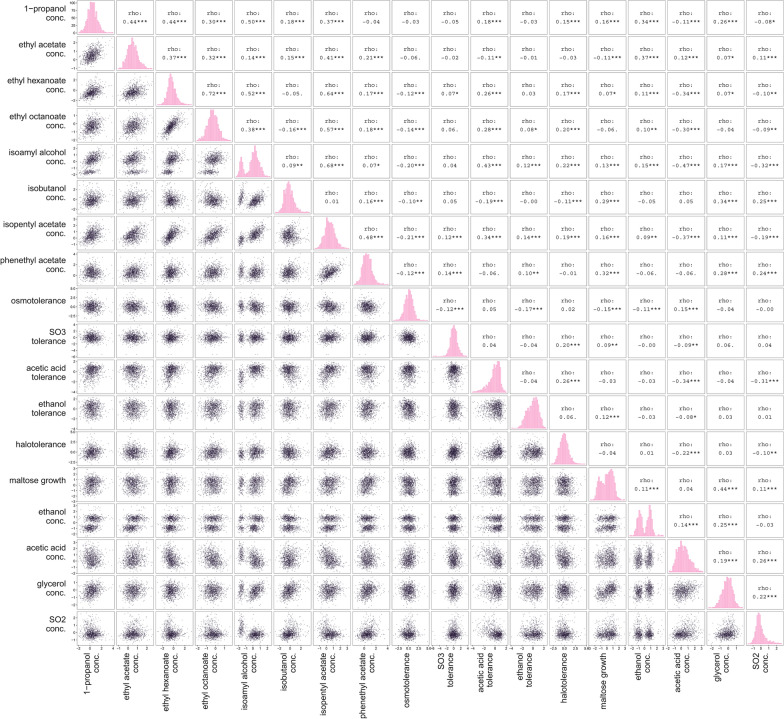
Phenotypic variance across 1125 F_6_ hybrids derived from a cross between *S. cerevisiae* strains RM11-1a and YJM975α [34]. Each of the 987 hybrids that did not display a growth defect on maltose was tested in conditions mimicking industrial beer fermentation (16^o^P at 20 °C for 7 days), after which concentration of primary (ethanol) and secondary metabolites (glycerol, acetic acid, higher alcohols and esters) were determined for each sample. In addition, the growth of all 1125 F_6_ segregants was measured in the presence of high osmolarity (sorbitol), salt, acetic acid, ethanol, acetic acid and sulfite (SO3^2−^). Spearman rank correlations were calculated for each pair of phenotypes, rho values are indicated here with corresponding significance (*p* < 0.10, *p < 0.05, **p < 0.01, ***p < 0.001)

Most traits show a normal distribution within the population, a typical characteristic of complex phenotypes that are controlled by multiple different genetic loci (Fig. 1). For a few phenotypes, however, the F_6_ progeny shows a clear bimodal segregation, indicative of one major segregating QTL that has substantial influence.

One such QTL was the *LEU2* marker, a gene encoding beta-isopropylmalate dehydrogenase that catalyzes the third step in leucine biosynthesis which was deleted in RM11-1a to facilitate selection of progeny during the repeated rounds of crossing [34]. The absence or presence of *LEU2* drastically influences the progeny’s phenotypes, especially in the production of metabolites that are directly related to amino acid and nucleic acid metabolism: isoamyl alcohol, isoamyl acetate, isobutanol, 1-propanol, ethyl octanoate, ethyl hexanoate, acetic acid, and SO_2_ (Additional file 2: Table S1). Hence, the marker functions as an artificial QTL and serves as a positive control for the QTL pipeline. Perhaps unsurprisingly, the most prominent effect of the absence or presence of *LEU2* is the formation of isoamyl alcohol, which is directly related to the leucine biosynthetic pathway in which Leu2 is involved. Segregants that harbor *LEU2* produce 193% more isoamyl alcohol in average compared to strains lacking it (*p* < 0.001) (Additional file 1: Figure S1). Because the effect of *LEU2* on many phenotypes is particularly strong and partly obscures more subtle differences related to natural QTLs, the segregants lacking *LEU2* were omitted and only the 845 prototrophic F_6_ segregants were used for the subsequent QTL analysis.

### Variance in industrial traits is driven by both rare and common, coding and intergenic variants

**Fig. 2:**
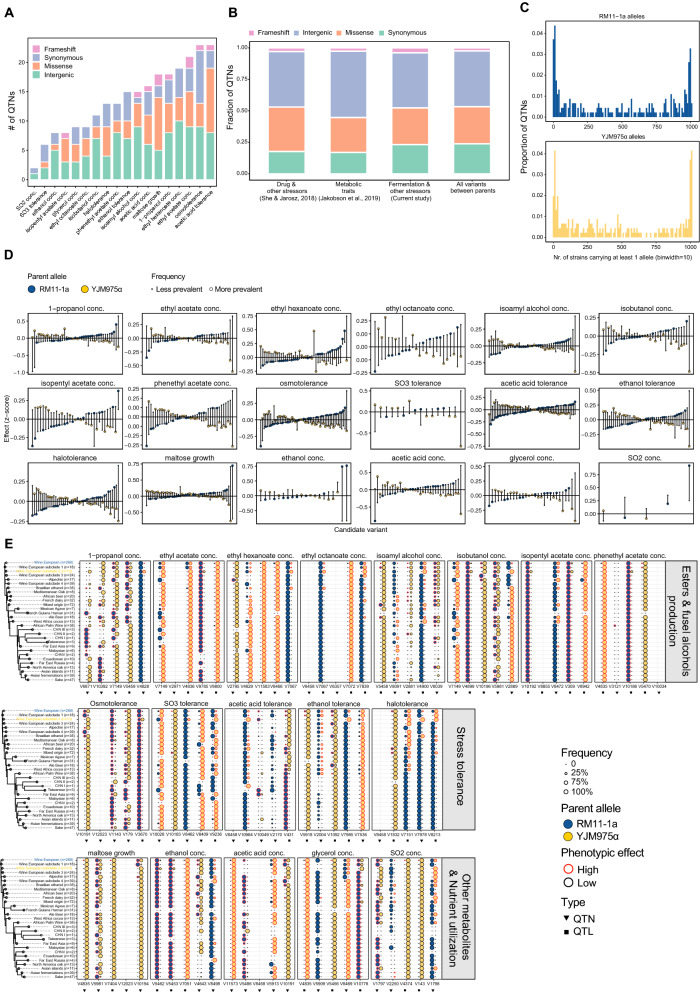
Overview of the identified QTLs underlying 18 industrially relevant phenotypes. **A** Number of causal variants of each functional class identified for each phenotype. **B** Comparison of the fraction of QTN of each functional class between studies employing the same cross; “Intergenic” includes all single nucleotide polymorphisms at non-coding regions, e.g., promoters, terminators, 5’ and 3’ UTRs, etc. **C** Histograms of the number of strains within the 1011 Genome Project yeast collection carrying at least 1 copy of the RM11-1a allele (top) and YJM975α allele (bottom) at the identified QTN across all phenotypes. **D** Phenotypic effect (as normalized z-scores) of the parental allele for each candidate locus (*N* = 678) and their prevalence in the 1011 sequenced *S. cerevisiae* strains [37]. The origin of the variants is indicated in blue (RM11-1a) and yellow (YJM975α), and the size of the point represents the frequency of the variant genotype in the 1011 sequenced *S. cerevisiae* isolates. **E** Phylogenetic distribution of the top 5 QTLs with strongest average effect on each trait (boxes) by lineage across the 1011 Genome Project yeast collection (phylogenetic tree adapted from [37], mosaic strains were excluded). For each site a distinction is made between QTN (triangle 1nt resolution) and QTL (square from 1 to 1000nt). Size of the circles indicates the fraction of strains within each lineage carrying at least 1 copy of the RM11-1a (blue) and YJM975α (yellow) allele at the specific locus. Red stroke indicates the parental allele with stronger effect on the trait. Lineage assignment is based on [37] and the number of strains is indicated next to the lineage name. RM11-1a and YJM975α lineages are colored blue and yellow, respectively

Analysis of 18 different industrially relevant phenotypes of the prototrophic F_6_ segregants yielded a total of 678 QTLs with *p* < 10^–5^ (Additional file 2: Table S2). Of the QTLs identified, 21 are small insertion or deletions (Indels) and 657 are single-nucleotide polymorphisms (SNPs). 254 QTLs could be unambiguously mapped to single-nucleotide resolution (QTN), and 58 could be mapped to single gene-level (size about 1 Kb). The QTNs responsible for these diverse traits included missense and intergenic (non-coding) variants, as well as synonymous and missense variants in coding regions (Fig. 2A). Roughly 43.7% of the QTNs locate in intergenic regions (*N* = 111), while synonymous and missense mutations each account for 23.2% (*N* = 59) and 29.1% (*N* = 74) of the QTNs, respectively. This distribution is very similar to the distribution of all variants between the founder strains (i.e., all polymorphisms between the two parental strains, irrespective of whether they are linked to a QTN or not; Fig. 2B). The presence of a large fraction of non-coding QTNs, including regulatory variants, is in line with the observation of former studies employing the same cross (Fig. 2B), where the complex traits tested were also fueled by polymorphisms of different molecular classes [34, 36].

As the majority of the industrially relevant traits included in the current study relate to fermentation performance, we asked whether we could identify a relationship between the presence of certain QTNs across the *S*. *cerevisiae* lineages, and if the ecology of the different strains are subject to enrichment that would offer evolutionary advantages, especially when a founder strain RM11-1a was isolated in a vineyard [4]. To elucidate the ecological relevance of our QTNs, we analyzed their distribution in a set of wholegenome sequences of 1011 *S. cerevisiae* strains, encompassing the full breadth of genetic and phenotypic diversity known for this species to date [37]. We removed 18 QTNs for which we were unable to consistently map the variant position across the 1011 strain collection. 54 QTNs are rare (present in < 1% of the 1011 strains): either unique to the parental strains, or shared by at most a small number of related strains (Fig. 2C). The contribution of low-frequency alleles confirms previous findings [38], and suggests that a substantial fraction of the missing heritability of complex traits might be explained by polymorphisms segregating at low frequency within populations and rare mutations might play a crucial role in modulating the phenotypic landscape within *S. cerevisiae* as previously reported [37].

By contrast, 78 QTNs are widely spread across the population and shared by 90%–99% of the strains in the collection (Fig. 2C).This is in line with the hypothesis of a single out-of-China origin for this species followed by geographical differentiation, human-associated admixture and several independent domestication events [37]. Next, we analyzed the distribution of all the identified QTLs over the *S. cerevisiae* natural diversity (as represented by the collection of 1011 sequenced strains). We included all candidate QTLs regardless of whether they were resolved to a single-nucleotide level. A proxy for allelic effects is shown in Fig. 2D as the difference in the mean of a given phenotype between the population of segregants containing either of the parental allele (i.e., distance between the two alleles in Fig. 2D). For clarity, only the 5 QTLs that were predicted to have the strongest effect size are shown in Fig. 2E for each trait. Both of the causal QTNs that were experimentally confirmed (i.e., V5462 in ethanol concentration and V11573 for acetic acid concentration; see below) appear to be rare mutations, accounting for less than 1% of the total genotyped alleles (Fig. 2E).

As might be expected, several QTLs are present across closely related lineages, indicating a shared evolutionary history and/or admixture between strains from different genetic backgrounds. For instance, the YJM975α allele of the V7151 QTL for halotolerance has been detected in most of the non-Asian lineages with the exception of the “Alpechin” and “French Guiana human” lineages. The RM11-1a allele of the V6871 QTL associated with 1-propanol production is mostly observed in Chinese, Taiwanese and Malaysian strains. Other QTLs are unique to a specific lineage and often segregate at low frequency within the population, suggesting a single emergence event in one or few related strains following lineage diversification. For instance, the RM11-1a allele linked to production of phenethyl acetate was detected only in the African Palm Wine lineage and the RM11-1a allele of the top ethanol tolerance QTL (V5918) was observed only in the Wine European clade and subclade 1. Such events can be caused by either drift or selection but disentangling this process in natural populations remains challenging [39].

Several QTLs are observed in multiple, non-related lineages indicating the presence of multiple emergence events or admixture/crossing. A prominent example is the RM11-1a allele of V10049 QTL for acetic acid tolerance, present in the “Far East Asia” and “Taiwanese” lineages together with the Wine European clade and subclade 2. The presence of independent emergence events across lineages, might hint at parallel evolution and positive selection, even if neutral drift can never be formally excluded. Interestingly, even though both founder strains are included in the 1011 genome collection (RM11-1a labeled in yellow and YJM975α blue in the respective clade, Fig. 2E), there are multiple alleles without a single appearance across the whole collection (e.g., V2971 from ethyl acetate and V8458 of halotolerance), suggesting novel variants arose in the founder strains used in this study. Moreover, we observed that 22% of the QTLs (*N* = 147) are multiallelic across the 1011 genome collection, i.e., show alternative alleles other than the two genotypes found in the parental strains that were used in the cross.

It is tempting to use methods like the McDonald–Kreitman test to further investigate whether any of the QTNs show signs of positive or negative selection. However, these methods need to be approached with care, since they assume difference in functional importance between synonymous and non-synonymous mutations [40, 41]. It is becoming increasingly clear that causal variants can encompass a wide array of molecular variation including synonymous and intergenic mutations [34, 36]. The level of polymorphism within species and the divergence is also heavily influenced by demography and the local adaption which varies from lineage to lineage and very little is currently known [41]. In a normal case, the stronger representation of a rare allele in certain lineages may hint to a selection or random genetic drift at play. However, certain phylogenetic clades, like the European Wine lineage, show more rare alleles. After experiencing a strong domestication bottleneck, the wine yeasts clade went through population expansion and accumulated many rare variants [37, 42]. In addition, although the mosaic strains have been removed from our phylogenetic analyses, many strains might still be the result of ancient admixture which can mislead assumption of parallel evolution if not accounted for.

### Experimental confirmation of five selected QTLs

Compared to the common procedures for QTL mapping in yeast, such as pooled segregant analysis, our pipeline identifies QTLs at the nucleotide-level (*N* = 254), single gene-level (size about 1 kb; *N* = 58) as well as larger genetic loci up to 5 kb (*N* = 106), which sometimes comprise multiple genes. Still, even these larger loci are considerably smaller than those typically identified in regular QTL approaches where QTL regions are often longer than 10 kb, making it relatively easier to predict which specific variant positions in the region are driving the phenotypic effect.

**Fig. 3:**
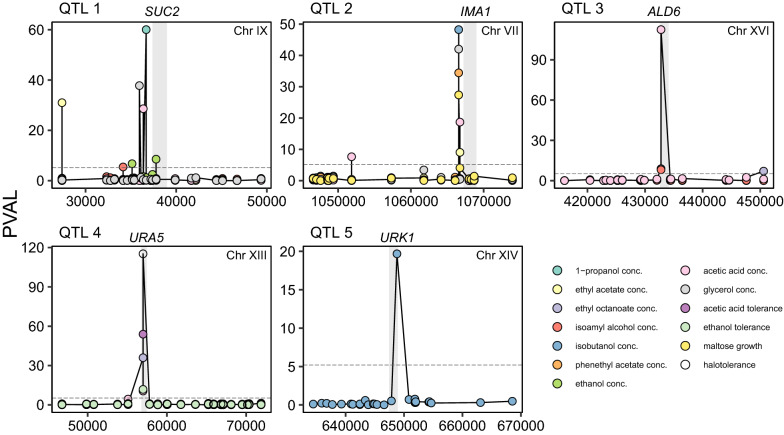
Genome-wide mapping identifies five candidate loci at single-nucleotide resolution that are predicted to affect multiple industrially relevant phenotypes. PVAL (− log_10_ transformed *p*-value from the forward selection) and the chromosomal position are shown for the variants centered around each candidate QTL (indicated in the grey rectangle). The threshold for selecting candidate variants is shown as dotted line (PVAL = 5.2)

To confirm the accuracy of our QTL mapping, we selected five QTLs that are predicted to affect multiple industrially relevant phenotypes for further investigation (Table 1 and Fig. 3). Four QTLs were selected because they are pleiotropic, linking to a broad range of different traits, from the production of primary metabolites such as ethanol, glycerol and acetic acid to secondary metabolites including valuable compounds like 1-propanol, ethyl acetate as well as tolerance towards salt and acetic acid (Fig. 3). An additional QTL was selected for its association with the production of isobutanol, which is not only an aroma-active compound that contributes to the flavor of fermented products, but is also being explored as a promising second-generation biofuel [43]. For each of these QTLs, we followed a similar strategy to identify the causative alleles or mutations and their respective phenotypic effects as described in deeper depth in “Materials and methods” section.

**Table 1:** QTLs called by forward selection regression﻿. List of QTLs with an influence on fermentation parameters﻿, the production of extracellular metabolites and volatile secondary metabolites (Additional files 3, 4, 5)

QTL	Trait	Chromosome	Start [bp]	End [bp]	PVAL range	Candidate genes within
*SUC2*	1-Propanol conc	IX	27,399	36,687	6.7–37.8	*NIT1*
	Ethyl acetate conc					*SUC2*
	Isoamyl alcohol conc					*YIL166C*
	Ethanol conc					*YIL169C-Gene*
	Acetic acid conc					
	Glycerol conc					
*IMA1*	Ethyl acetate conc	VII	1,066,563	1,066,735	7.3–42.0	*BIO2*
	Isobutanol conc					*IMA1*
	Phenethyl acetate conc					
	Acetic acid conc					
	Glycerol conc					
	Maltose growth					
*ALD6*	Ethyl octanoate conc	XVI	432,771	434,477	8.1–112.4	*ALD6*
	Isoamyl alcohol conc					
	Acetic acid conc					
*URA5*	Ethyl octanoate conc	XIII	56,990	56,990	10.5–115.2	*URA5*
	Acetic acid conc					
	Acetic acid tolerance					
	Ethanol tolerance					
	Halotolerance					
*URK1*	Isobutanol	XIV	646,585	648,789	8.2	*URK1*

### Variation in *SUC2* causes differences in the production of alcohol and various other metabolites

**Fig. 4:**
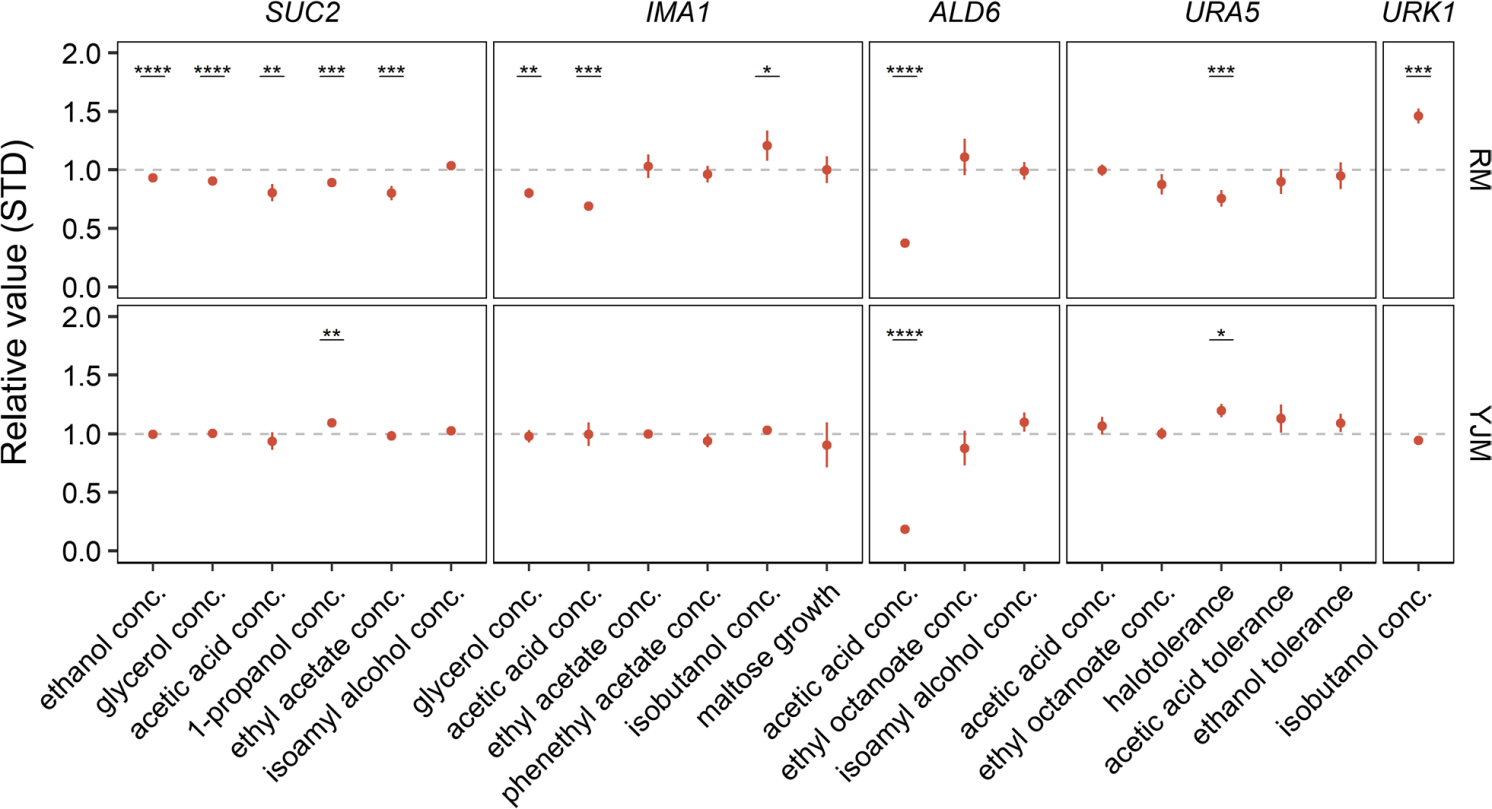
Effect of deleting genes that overlap with predicted QTLs on the mapped traits (Table 1) in both haploid strains RM11-1a (RM) and YJM975α (YJM). The trait value of the wild-type strain is set at 1. Each point is represented as the mean ± STD of at least three biological replicates after normalization against the mean of the respective wild-type strain for every phenotype (dotted line). *p*-values are indicated by asterisk symbols; *: *p* ≤ 0.05, **: *p* ≤ 0.01, ***: *p* ≤ 0.001, ****: *p* ≤ 0.0001

One hugely important industrial trait is the ability to convert all available fermentable sugars into ethanol. For this phenotype, we identified 21 QTLs. Among the candidate QTLs, *SUC2* stands out because of two reasons. First, this QTL was also mapped to other traits with high significance, including the production of 1-propanol, ethyl acetate, acetic acid, and glycerol (Additional file 2: Table S2). Second, it encodes sucrose invertase, the enzyme that catalyzes the first step in sucrose metabolism, namely the hydrolysis of sucrose into glucose and fructose. Hence, it is tempting to speculate that variation in *SUC2* could lead to different efficiencies in sucrose metabolism, which would in turn explain the observed differences in the production of primary (ethanol) and certain secondary metabolites. In line with this hypothesis, deletion of *SUC2* in strain RM11-1a led to 6.5% reduction (*p* < 0.0001) in the final ethanol concentration in the sample, while no effect was observed in strain YJM975α (Fig. 4). Similarly, *SUC2* deletion also led to reduced formation of 1-propanol (− 10.8%; *p* ≤ 0.001), ethyl acetate (− 17.9%; *p* ≤ 0.001), acetic acid (− 16.5%; *p* ≤ 0.01), and glycerol (− 10.8%; *p* ≤ 0.0001) in RM11-1a, but had no effect in the strain YJM975α, except for the formation of 1-propanol (+ 8.6%; *p* ≤ 0.01) (Fig. 4). As the deletion of *SUC2* in YJM975α did not result in significant changes in most of these metabolites, the *SUC2* allele of YJM975α is likely to be a loss-of-function allele, rendering the cells with inferior efficiency in sucrose metabolism as compared to that of RM11-1a.

**Fig. 5:**
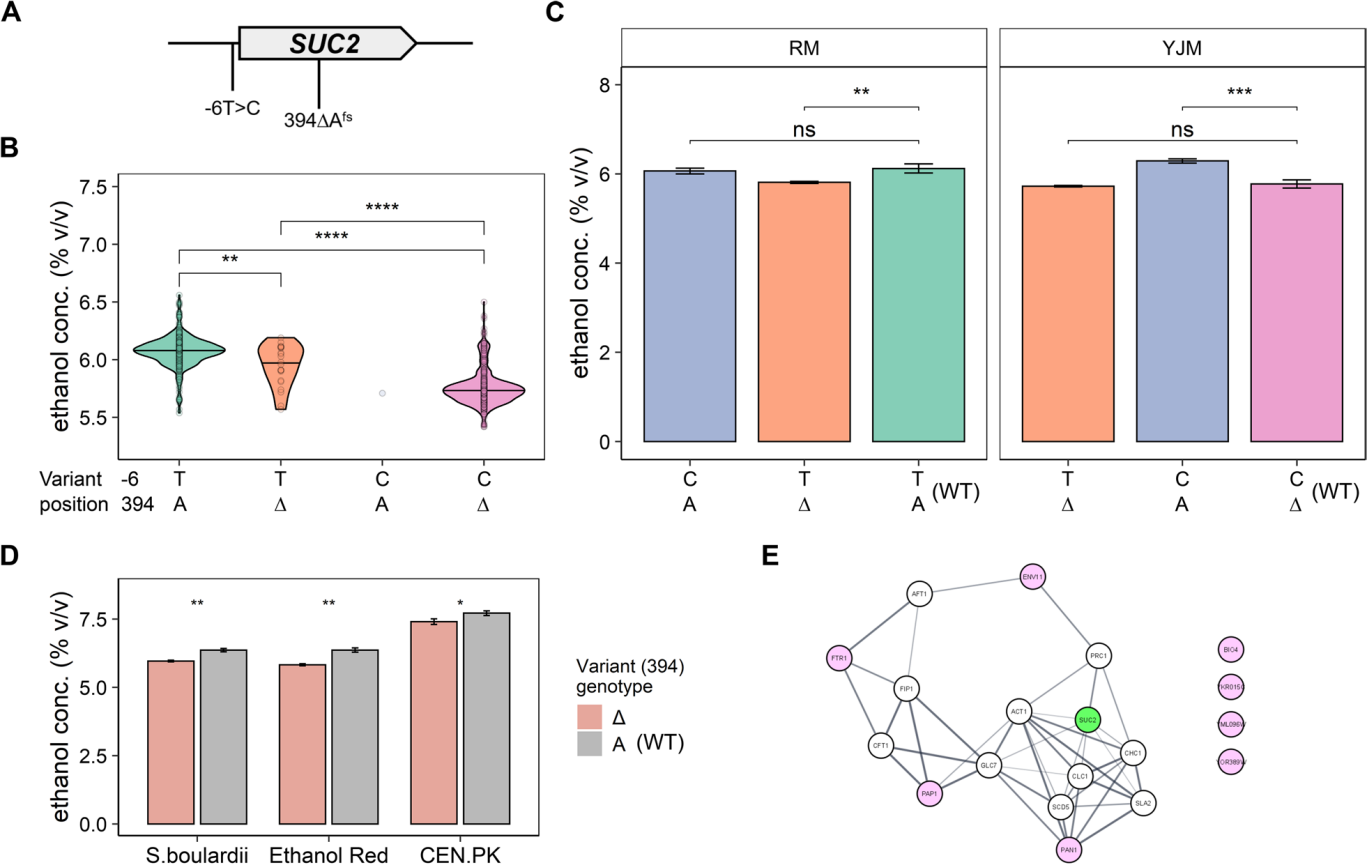
Identification of the causal variant of reduced ethanol production in the *SUC2* locus**. A** Candidate variants in *SUC2*. **B** Meiotic crossovers within the *SUC2* locus in the F_6_ segregants. Swapping the intergenic variant (-6) yields minor phenotypic effect, whereas swapping the true causal variant (394) yields the same major effect as swapping the entire haplotype block. **C** Ethanol concentration at the end of fermentation (16^o^P) of the wild type (WT) strain RM11-1a and YJM975α and the respective variant-swapped mutants. **D** Ethanol concentration at the end of fermentation (16^o^P) of the wild-type strain *S*. *boulardii*, Ethanol Red and CEN.PK and the *SUC2*^394∆^ variant mutant. **E** Interaction network of *SUC2* (green node) and genes whose coding sequence are altered by variants that were identified for ethanol concentration phenotype (pink node). The thickness of the edges represents the confidence score associated with the interaction as determined by STRING. Data are shown with mean ± STD; *P*-values are indicated with the level of significance (ns: not significant, *: *p* ≤ 0.05, **: *p* ≤ 0.01, ***: *p* ≤ 0.001, ****: *p* ≤ 0.0001)

Next, we attempted to identify the exact causative genetic variation in *SUC2* that is responsible for the observed phenotypes. Our QTL pipeline highlighted one frameshift variant in the ORF of *SUC2* gene (*SUC2*^394∆^) with high significance (PVAL > 5.2) from several traits. However, since the mapping analysis cannot unambiguously distinguish this variant from a nearby variant located at position − 6 in the promoter of *SUC2*, we also included this variant for our validation (Fig. 5A). Although segregants inheriting the entire haplotype block (both *SUC2*^−6A^ and *SUC2*^394A^) from RM11-1a produced higher amounts of ethanol (Fig. 5B), we confirmed that this frameshift mutation is causative for the observed reduction in ethanol production (Fig. 5C). This variant rests immediately at 5’ of Suc2’s catalytic site [44], and might influence the affinity of the enzyme, as residual sucrose was detected in the finished fermentation sample (Additional file 1: Figure S3). Moreover, it has an opposing effect on the formation of acetic acid and glycerol (Additional file 1: Figure S4), showing its pleiotropy. The fact that the truncated *SUC2* allele affects multiple phenotypes is perhaps not surprising, as mutations in this gene affect the total amount of carbon that can be metabolized, thus influencing primary and secondary metabolite production, as well as other traits that may be indirectly linked to growth and metabolism.

To test if the truncated *SUC2* allele leads to reduced ethanol production in other strains containing an intact *SUC2* allele, we introduced the *SUC2*^*394∆*^ frameshift variant in three diverse industrially relevant strains: (i) Ethanol Red (an industrial bioethanol strain; (ii) CEN.PK (a common chassis strain for heterologous production of high-value compounds) and (iii) *S*. *cerevisiae var. boulardii* (a strain often referred to as *S*. *boulardii*, and commercially used as probiotic). Introduction of the frameshift variant indeed led to reduced ethanol formation in all three strains (Fig. 5D), substantiating the effect of a truncated *SUC2* allele on ethanol formation. Next, we investigated whether other candidate QTLs that were mapped to ethanol production would interact with *SUC2*, however, we did not observe a clear network among these mapped QTLs for ethanol production (Fig. 5E).

### YJM975α contains incomplete *IMA1* locus, causing reduced maltose utilization and altered formation of certain metabolites

Within QTL2, linked to several traits such as glycerol and acetic acid production as well as maltose utilization, several polymorphisms were identified in the intergenic region between *BIO2* and *IMA1* genes (approximately 2.2 kb) that are located within the subtelomeric region of chromosome VII (Fig. 3C). While deletion of *BIO2* in either of the strain RM11-1a and YJM975α did not affect any of the phenotypes to which the QTL was linked (data not shown), the deletion of *ima1* in RM11-1a led to reduced production of glycerol (− 21.7%; *p* ≤ 0.01) and acetic acid (− 30.8%; *p* ≤ 0.001), and increased production of isobutanol. By contrast, *IMA1* deletion in strain YJM975α did not result in any significant changes (Fig. 4).

The *IMA1* gene encodes for the major isomaltase required for isomaltose utilization, which also exhibits α-1,2 glucosidase activity on sucrose and kojibiose [45, 46]. Near the *IMA1* locus, one intergenic variant (*IMA1*^+*659G*>*C*^) was predicted to influence several traits with strong significance (PVAL > 50). Comparison of the *IMA1* locus between strain RM11-1a and YJM975α revealed one missense mutation (*IMA1*^1007A>T^), which has been reported to have a deleterious effect on the growth on raffinose, sucrose and maltose [36]. Yet, swapping either of the two variants between the two strains did not lead to any significant change in the phenotypes that were observed in the *ima1* deletion mutant (Additional file 1: Figure S5). However, a closer evaluation of the YJM975α genome sequence revealed that it lacks approximately 8 kb of the genomic region directly upstream of *IMA1*, including part of the 5’end of *IMA1* (207 bp) as well as the entire coding regions of *MAL13*, encoding the activator protein that activates the permease and hydrolase when substrate is present, and *MAL11*, encoding the maltose/isomaltose permease [47]. This 8-kb deletion segregates in the F_6_ progeny (Additional file 1: Figure S6A) and correlates with the growth on maltose (Additional file 1: Figure S6B), with segregants containing the intact *MAL-IMA1* locus showing more efficient growth on maltose, indicating that this structural variation is at the heart of this QTL.

### Variation in *ALD6* drives changes in acetic acid production

**Fig. 6:**
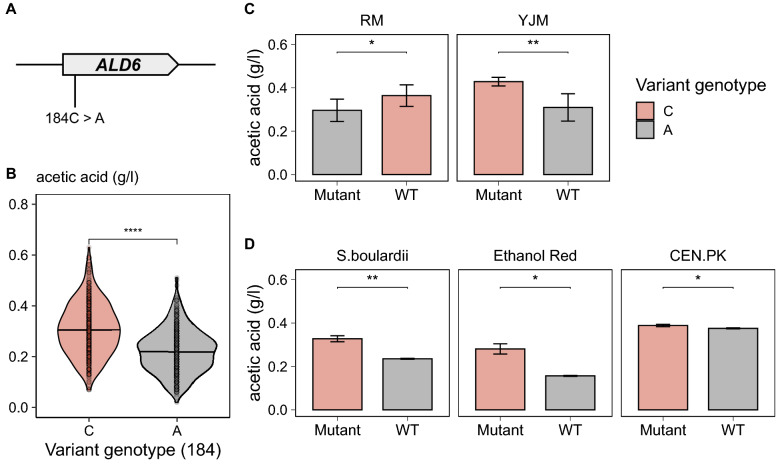
Identification of the causal variant underlying differences in acetic acid production **A** Candidate variants in *ALD6*. **B** Meiotic crossovers within the *ALD6* locus in the F_6_ segregants. **C** Acetic acid production of the variants swapped in the parent strain RM11-1a and YJM975α, and **D** in *S*. *boulardii*, Ethanol Red, and CEN.PK. Error bars represent standard deviation of 3 biological replicates. *P*-values are indicated with the level of significance (*: *p* ≤ 0.05, **: *p* ≤ 0.01, ***: *p* ≤ 0.001, ****: *p* ≤ 0.0001)

For QTL3, deletion of *ALD6* resulted in significantly reduced acetic acid production in both parental strains, by 63.2% in RM11-1a (*p* ≤ 0.0001) and by 81.7% (*p* ≤ 0.0001) in YJM975α (Fig. 4). *ALD6* encodes cytosolic aldehyde dehydrogenase, an enzyme required for the conversion of acetaldehyde to acetate. Our QTL pipeline linked the changes in acetic acid production to a missense variant (*ALD6*^*184A*>*C*^) located in the *ALD6* ORF (Fig. 6A). Segregants inheriting the haplotype from RM11-1a (*ALD6*^*184C*^) produced higher amounts of acetic acid compared to the ones containing the YJM975α allele (*ALD6*^*184A*^) (Fig. 6B). Swapping the specific alleles between RM11-1a and YJM975α led to a 22.3% (*p* ≤ 0.05) reduction in acetic acid formation in RM11-1a, and a 22.0% (*p* ≤ 0.001) increase in YJM975α, confirming that this variation is indeed the driver in this QTL (Fig. 6C).

To assess the background-specificity of the mutation, we introduced the *ALD6*^*184C*^ variant in strain Ethanol Red® and strain CEN.PK as well as in strain *S. boulardii*, which all carry the *ALD6*^*184A*^ allele. Especially for this last strain, increased acetic acid production would be hugely beneficial, since its probiotic effect is at least partly attributed to the production of acetic acid, which can affect the growth of other microbes in the gastrointestinal tract [48]. Introduction of the mutation indeed led to increased acetic acid formation in all three strains, most notably in Ethanol Red (79%) and *S. cerevisiae var. boulardii* (39%), showing that some of the QTLs identified in this study could serve as a basis to further improve industrial strains (Fig. 6D).

### Some QTLs can be linked to a gene, but not an exact allele

Deletion of *URA5* led to salt sensitivity in strain RM11-1a, while improving salt tolerance in strain YJM975α (Fig. 4), suggesting that *URA5* is the causal element in QTL4 for the differential halotolerance between the two strains. Further analysis identified a missense variant within *URA5* (*URA5*^*266G*>*T*^), which encodes for the major orotate phosphoribosyl transferase (OPRTase), catalyzing the fifth enzymatic step in de novo biosynthesis of pyrimidines [49]. However, despite several attempts we could not swap the missense variant between the parent strains and were therefore unable to confirm whether this point mutation drives the QTL. In addition to halotolerance, the *URA5-*related QTL is also linked to other traits such as ethanol tolerance (Fig. 3). It is likely that this pleotropic effect is exerted through the biosynthetic pathway of pyrimidines. Indeed, *URA5* catalyzes the step upstream of *URA3* which has already been connected to ethanol tolerance as the causal QTL in a cross between a bioethanol production strain and a laboratory strain [30].

Similarly, for QTL5, deletion of *URK1* resulted in significantly increased production of isobutanol by 47.2% in RM11-1a (Fig. 4). *URK1* encodes pyrimidine kinase, an enzyme involved in the deoxyribonucleotide salvage pathway. Interestingly, the deletion of *URK1* in Ethanol Red and CEN.PK also led to increased production of isobutanol, while no effect was observed in *S*. *boulardii* (Additional file 1: Figure S7B), suggesting that there might be complex background interactions that further influence this trait*.* Mapping analysis identified two missense variants in the ORF of *URK1* (*URK1*^*412A*>*C*^ and *URK1*^*1358G*>*A*^). However, swapping either of the two variants between strain RM11-1a and YJM975α did not lead to any significant change in the production of isobutanol (Additional file 1: Figure S7A).

## Discussion

Our study identified 678 putative QTLs that are predicted to drive differences in various industrially relevant phenotypes, including resistance to various stress factors and the production of primary and secondary metabolites. Further analysis of 5 QTLs showed that in each case, we were able to link a certain gene located within the QTL to some or all phenotypes that are influenced by the QTL. In two cases, the analysis pipeline correctly predicted specific polymorphism at single-nucleotide level as the driver of the QTL. In another case, the QTL proved to be linked to a large segmental deletion. In the two remaining cases, we were able to link a specific gene to the respective traits, but unable to pinpoint the exact causative mutation.

### *LEU2* auxotrophy influences the formation of fusel alcohols and esters

The use of auxotrophic marker in strain construction rests on the assumption that in medium where the auxotrophy is complemented by supplementation of a specific compound, the phenotypic effect is minimal. However, this assumption may not hold true when the auxotrophically required compounds interfere with the biosynthesis of other metabolic compounds and/or influence the expression of specific genes [50]. We also observed this in our data, where the specific growth rate of wild-type *S*. *cerevisiae* is reduced when only leucine is supplemented in the synthetic medium without the addition of isoleucine or valine, which is in line with the previously reported influence of leucine on the biosynthesis of the other two branched-chain amino acids [51]. Apart from the impact on physiology, amino acid autotrophy has also already been linked to a number of industrially relevant traits. For example, *URA3* was identified as the causal QTL for ethanol tolerance in a cross between a bioethanol production strain and a laboratory strain [30]. The same auxotrophy also leads to the difference in maximal ethanol accumulation capacity between a sake and a laboratory strain [52]. Similarly, leucine autotrophy has been suggested to affect the cell’s resistance to rapamycin in the same set of segregants used in this study [34]. Here, we show that leucine auxotrophy imposes a strong effect on fermentation phenotypes, and specifically aroma formation, because metabolism of leucine in yeast is tightly linked to the formation of various flavor-active compounds [53], including organic acids, aldehydes, and higher (fusel) alcohols [54]. Several of these higher alcohols are currently explored as biofuels [55], but they are also the main precursors of acetate esters, which are important contributors to the flavor and aroma of alcoholic beverages [56, 57]. In particular, *LEU2* encodes for beta-isopropylmalate dehydrogenase, involving in the conversion of α-ketoisovalerate (KIV) to α-ketoisocaproate (KIC) which is a direct intermediate in the biosynthetic pathway of isoamyl alcohol. As a result, the production of isoamyl alcohol and its derivative ester isopentyl acetate are prominently affected in the F_6_ progeny by the segregation of leucine auxotrophy (Additional file 1: Figure S1). The fundamental impact of leucine auxotrophy on the traits measured in the current study highlights how changes in the expression of a single auxotrophic marker can resonate throughout the entire metabolic network and lead to an altered pool of metabolites. Therefore, great care should be exercised in the design of the experimental setup to mitigate possible drawbacks from the application of these auxotrophic markers (see also [50]).

### Abundant pleiotropic interaction among identified QTLs

Pleiotropy refers to the effect of a particular allele on more than one phenotype. Our data indicate that pleiotropy is common across traits examined in this study. At the molecular level, roughly 39% (*N* = 267) of the QTLs are linked to more than one trait (Additional file 2: Table S3). This may also at least partly explain why certain phenotypes are correlated (Fig. 1). We define a pleiotropic QTL as a variant that is linked to more than one phenotype, or when several variable sites within one QTL region (with a maximal dimension of 10 kb) are linked to different traits. This is particularly important for the identification of the segmental deletion at the *IMA-MAL* locus as well as the precise validation of the causal frameshift variant located in *SUC2*. In the former case, as the deleted genomic region prevents association of precise variants within this locus, several intergenic variants close to the deleted locus were linked to multiple traits without nucleotide-level resolution. In the latter case, the close vicinity of the frameshift variant *SUC2*^*394∆A*^ and an intergenic variant that locates 400 bp away prevents frequent recombination between the haplotype block in the F_6_ segregants, leading to multiple candidate variants that could not be unambiguously distinguished.

Swapping of the *SUC2*^*394∆A*^ allele between the founder strains results in a concerted change in the production of ethanol, glycerol, and acetic acid. This interconnection is in line with previous reports [58–60]. One possible explanation that has been suggested is that while alcoholic fermentation is redox neutral, the biomass formation generates surplus NADH, requiring other pathways for the regeneration of NAD^+^ to maintain flux through glycolysis. In response, cells synthesize glycerol to regenerate NAD^+^, however, excess NAD^+^ may be formed, which is then balanced through the formation of acetic acid from acetaldehyde to convert NAD^+^ back to NADH that functions as redox sink.

### Structural variation affects multiple fermentation-relevant traits

In all-malt brewer’s wort, maltose typically accounts for 60% of the total fermentable sugar for the yeast and efficient maltose utilization is therefore crucial for beer production. Maltose metabolism requires 3 key genes, encoding maltase (MALS), a transporter for maltose (MALT) and a positive regulator (MALR) [61]. These 3 genes typically cluster together in the genome in subtelomeric regions, and most genomes harbor several such gene clusters, scattered across different chromosomes [46, 62]. In some cases, the elevated rate of mitotic and meiotic recombination in the subtelomeric region may also lead to chromosomal rearrangements in these *MAL* loci. For instance, the impaired growth phenotype on maltose is linked to the deleted genomic region (ca. 8 kb) at the *IMA1*-*MAL1* locus in the genome of YJM975α. This structural variation highlights the relevance of the copy number of the *MAL* (maltose) gene with utilization of maltose, which is in accordance with the findings of [1], where yeast domestication led to more efficient fermentation of specific carbon sources like maltose through mutations and duplications of the *MAL* genes [1]. In addition to maltose growth phenotype, the missing locus also links to other traits, including the production of ethyl acetate, isobutanol, phenethyl acetate, acetic acid, and glycerol (Additional file 2: Table S2). The paramount impact of maltose utilization on the production of metabolites was already anticipated at the start of the project, and all F_6_ segregants were therefore propagated in YPMaltose for 3 days prior to the main fermentation to detect major growth defects. The profound pleiotropic effect of this locus calls for a more stringent screening scheme, which would inevitably be at the cost of reducing mapping resolution.

While absence of the entire *IMA1-MAL1* locus is linked to impaired maltose utilization, deletion of the *IMA1* locus by itself also leads to multiple phenotypes in RM11-1a, including the reduced formation of glycerol, acetic acid and isobutanol (Fig. 4). The pleiotropic effect of *IMA1* may be explained by the substrate specificity of the corresponding Ima1 enzyme, which shows activity towards sugar oligomers with α-1,6- or α-1,2-glycosidic linkages, such as isomaltose, sucrose and dextrin [45, 63]. Specifically, *IMA1* has been suggested to have a minor (yet relevant) activity towards sucrose in the absence of *SUC2*, which encodes the major sucrose invertase [64]. Although these α-glucosides are present in relatively small quantity in the wort [65], the assimilation of these sugars impacts beer quality and affects the production of flavor-active metabolites. For instance, significant differences in volatile profiles were obtained as different carbohydrates were fermented by yeast [66].

### Prevalence of the identified causal QTNs across a diverse strain collection

The distribution of the different QTNs that were identified in this study closely resembles the distribution of all SNPs across *S*. *cerevisiae* strains (Fig. 2). This might suggest that many QTNs only have relatively minor, near-neutral effects and that are not under strong (negative or positive) selection. Still, our analyses show that a few variants, such as for example the *SUC2*^*394∆A*^ frameshift mutation, do have major phenotypic effects. From an ecological standpoint, the *SUC2*^*394∆A*^ frameshift allele renders cells with reduced ability to utilize a carbon source (i.e., sucrose), so such deleterious alleles are predicted to be rare when natural selection operates on the QTL. Recurring deleterious alleles is an important source of genetic variation which can result from de novo mutation or transfer of the alleles from a nearby population through hybridization or horizontal gene transfer. However, it is not trivial to classify a given mutation as negative because it might be deleterious in existing environments or genetic backgrounds but beneficial in other ecological or genetic contexts. As an example, the *ALD6*^*184A*>*C*^ variant resulted in elevated formation of acetic acid production, which can help suppressing growth of competing bacteria in complex microbial communities [48], but might be detrimental for the yeast’s fitness in man-made, pure-culture fermentation environments [67].

### Context-dependent QTL

Isobutanol is a promising second-generation biofuel [43], which is produced by yeasts in small amounts through the degradation of valine via the Ehrlich pathway [54], and multiple metabolic engineering approaches have been proposed to boost production [68, 69]. Our results show that some QTLs also influence the natural production of isobutanol, and this led us to ask whether these could be used to increase the production of this metabolite. We verified *URK1* as the causal QTL for the production of isobutanol. Moreover, deletion of *URK1* resulted in similar phenotypes across different strains, suggesting the effect of this QTL is not context-specific and could be used to boost isobutanol production in industrial strains. Urk1 is a uridine kinase involved in the pyrimidine salvage pathway, which is responsible for the biosynthesis of pyrimidines required for the synthesis of nucleic acid and amino acids [70]. However, a direct link between Urk1 and isobutanol production has never been reported.

## Conclusions

In the current study, we identified 678 candidate variants for 18 industrially relevant traits using an inbred yeast population consisting of 1,125 F_6_ progeny of a cross between two strains, RM11-1a and YJM975α. Experimental validation confirmed the contribution of five genetic loci, of which two were pinpointed to the single-nucleotide level. The identified loci consisted of both coding and intergenic regions, and comprised a broad range of different types of mutations, ranging from structural variation to InDels and SNPs. Interestingly, the effect of some industrially relevant QTLs was consistent in different genetic background. For example, transferring the *ALD6*^*184A*>*C*^ QTN in a probiotic strain increased its acetic acid formation (and thus possibly also its probiotic effect), while deletion of *URK1* led to increased isobutanol production in an established biofuel strain. On the other hand, we also show that many of the loci affect multiple phenotypes, which implies that engineering these QTLs into a strain will likely also result in multiple changes, a factor that has not received sufficient attention in studies promoting gene editing of industrial yeasts. Together, our results demonstrate the effectivity of the described approach to detect causal variants for complex traits and open new avenues for optimizing strains in a broad range of biotechnological applications.

## Materials and methods

### Yeast strain

The yeast strains used and constructed in the study are listed in Additional file 2: Table S4. The parent strain RM11-1a and YJM975α as well as their 1,125 F_6_ haploid progeny were obtained from Dr. Jarosz group [34]. *Saccharomyces boulardii* was isolated from lyophilized cells that is available in the market as probiotics in capsules (BIOCODEX, BENELUX). The strains were routinely maintained on solid YPD medium containing 10 g L^−1^ yeast extract, 20 g L^−1^ peptone, 20 g L^−1^ glucose, and 15 g L^−1^ agar. Frozen stocks of all strains were maintained at − 80 °C using a glycerol-based storage medium (20 g L^−1^ Bacto peptone, 10 g L^−1^ yeast extract, 20 g L^−1^ glucose, 250 mL L^−1^ glycerol).

### General molecular biology and microbiological techniques

Genomic DNA extraction from yeast was performed using phenol–chloroform–isoamyl alcohol (PCI) according to the method described by [71]. Plasmids were isolated from *E*. *coli* DH5α cells from overnight cultures in lysogeny broth (LB) containing 10 g L^−1^ peptone, 5 g L^−1^ yeast extract and 10 g L^−1^ NaCl (pH 7.0) with 100 mg L^−1^ carbinicilin by using the Qiagen Miniprep Kit (Qiagen, Germany). Transformation of yeast cells with plasmids as well as PCR-amplified DNA fragments for genomic integration was performed using LiAc/PEG method described by [72].

### Lab-scale fermentation in wort

Yeast pre-cultures were inoculated overnight at 20 °C in test-tubes containing 3 mL of 10 g L^−1^ yeast extract, 20 g L^−1^ peptone, 40 g L^−1^ maltose medium (YPMal). After 16 h of incubation, 1 mL of the pre-culture was used to inoculate 50 mL of the YPMal medium in 250-mL Erlenmeyer flask and propagated in the same conditions as the pre-culture for 3 days. Notably, 10% of the segregants showed growth defects during pre-culturing and thus were exempted from further fermentation. The propagated cells were then used for inoculation of the fermentation medium, i.e., 16° Plato (16^o^P) wort prepared by in-house brewery at a pitching rate of 10^6^ cells mL^−1^. A blank wort medium was included in each batch of fermentation. Fermentations were performed in 250-mL Schott bottles with a water lock placed on each bottle and stirred at 150 rpm for 7 days at 20 °C. Weight loss was monitored daily to follow the progress of fermentation. After 7 days, the fermentations were terminated on ice to minimize evaporation of volatile compounds and sampled for analytical analysis. Fermentation of all segregants was individually performed only once.

### Analytical methods

Quantification of yeast aroma production was carried out using headspace gas chromatography coupled with flame ionization detection (HS-GC-FID; Shimadzu, Japan). The GC was calibrated with 8 important aroma compounds, including isobutanol, isoamyl alcohol, ethyl acetate, ethyl hexanoate, ethyl octanoate, isoamyl acetate and phenethyl acetate, using 2-heptanol as the internal standard. Specifications of the GC system and the sample preparation are as described by [73]. Ethanol measurements were performed with the Alcolyzer Beer DMA 4500 M (Anton Paar, Austria). Filtered samples (0.15 mm paper filter) were measured for the level of glycerol, acetic acid and sulfite using Thermo Scientific Gallery discrete photometric analyzer (Thermo Fisher Scientific, USA). Sugar concentrations were determined by Dionex Liquid Chromatography (Thermo Fisher Scientific, USA), which was calibrated for maltose, sucrose, glucose and fructose using raffinose as the internal standard.

### Yeast phenotyping

F_6_ segregants taken from frozen stock were pinned on solid YPD medium using a Singer ROTOR robotic pinning instrument, with which cells were subsequently transferred to various solid media (YPD with stress agents as indicated, carbon source is 20 g L^−1^ glucose if not otherwise indicated) for phenotyping and incubated at 30 °C for 48 h. Cells were duplicated on a blank YPD plate (containing no additional stress/agent) for each growth assay to normalize for inherent growth differences. Growth was monitored daily in 1536-spot format by scanning the plates; colony size was quantified by using the programming language R (www.r-project.org) with package R/gitter v1.1.1 [74]. Prior to data analysis, cell growth was normalized by equating colony sizes of the trait to that of the corresponding blank plate.

### Mapping of the variants

Phenotypical measurements were corrected for batch effects for visualization purposes by performing stepwise regression for each phenotype with genotypes and fermentation batches as predictors (*p* cutoff = 0.01). Resulting coefficient estimates were multiplied with measurements from the corresponding batch. Quantitative trait nucleotide (QTN) scores for each phenotype were calculated using the pipeline developed by [34] (*p* cutoff = 0.01, false discovery rate cutoff log(*p*) = 5.2). Due to the drastic effect of the *LEU2* deletion on general fermentation performance, all subsequent analyses of the traits that are known to be highly linked to *LEU2* were performed separately on the segregants with and without the gene.

During the propagation step prior to fermentation, approximately 15% of the progeny showed compromised growth on maltose (OD_600_ < 1.0 after 3 days of cultivation in YPMaltose). As this growth defect leads to only partial consumption of the available carbon, which in turn causes large deviations in metabolite production that obscure some of the more subtle differences induced by other QTLs. Segregants showing growth defects on maltose were therefore excluded from experiments aimed at characterizing metabolite production during fermentation, and only the remaining 758 segregants that did not exhibit a growth defect on maltose were included in the analysis pipeline. A set of other phenotypes, most notably resistance to stress factors, were tested in rich YPD medium with glucose as carbon source. These phenotypes are therefore not influenced by maltose consumption, and the data for all 845 prototrophic F6 segregants were used in the analysis pipeline.

### Experimental confirmation of five selected QTLs and variant replacements

Validation of the candidate variants was carried out in two steps. In a first step, we tested the contribution of several candidate genes located within the predicted QTLs to a given phenotype by checking the phenotypic effect of deleting the gene in both parental genetic backgrounds. Apart from their central location in the predicted QTL regions, the target genes were selected either because they contain non-synonymous variants between the parent strains RM11-1a and YJM975α and/or have a molecular phenotype (enzymatic activity or transcriptional activity) that could be linked to the specific phenotype under investigation. For some QTLs, we performed a second set of experiments where the parental alleles were swapped.

The locus harboring a candidate variant was deleted in both parent strains by genomic integration of a disruption cassette containing the nourseothricin (clonNAT) resistance gene (NatMX). The deletion cassette was obtained by PCR from the plasmid pV1382 (addgene, USA) using primers del_QTL_fw and del_QTL_rv (Additional file 2: Table S5). When phenotypic difference was observed between the constructed mutant and wild-type strain, the candidate variant was subsequently swapped between the parents via CRISPR–Cas9-mediated genome editing. To target each candidate variant, a unique guide RNA (gRNA_QTN_fw; gRNA_QTN_rv) containing plasmid was constructed based on pV1382 as the backbone (Additional file 2: Table S5). Repair fragments (100 bp) containing the parental genotype of each target variant was prepared by annealing primers RF_QTN_fw_parent and RF_QTN_rv_parent (Additional file 2: Table S5) with 50–60 bp extensions homologous to regions up- and downstream of the target locus. To swap the target QTN in the parent strains, the respective guide RNA plasmid and the repair fragments containing the genotype of the counterparts were co-transformed reciprocally. Transgenic strains were selected on YPD solid medium supplemented with 200 µg mL^−1^ ClonNAT. The correct constructs of the QTL deletion and QTN swap mutants were verified with PCR and/or Sanger sequencing using primers ver_QTL_fw and ver_QTL_rv (Additional file 2: Table S5).

### Genetic mapping for growth differences on maltose

Copy number and mutations on the MAL genes were identified by using the raw sequencing data from [34]. Briefly, raw reads were trimmed and filtered using Trimmomatic v.0.33 [75], clipping bases with quality score below 20 and discarding reads shorter than 30 bp. The quality of the resulting trimmed reads was assessed with FastQC v0.11.8 (http://www.bioinformatics.babraham.ac.uk/projects/fastqc/). Trimmed reads were aligned to S288C reference genome with the Burrows-Wheeler Aligner v.0.7.17 [76], and short variants were detected following the Genome Analysis Toolkit best practices, using GATK v4.0.11 [77]. After variants genotyping, the allelic information for each of the 1,152 samples were extracted using a custom python script. For each sample, *IMA1* long and short alleles were identified by assessing the *IMA1* locus and upstream region coverage (chromosome VII: 1,069,000–1,076,000 bp) with bedtools v2.29.0 [78]. All results were confirmed by carefully analyzing mapped reads tracks on genome browser IGV v2.4.16 [79].

### Analysis of QTL distribution across natural *S. cerevisiae* strains

The 1011Matrix.gvcf.gz, containing all SNPs and indels called at the population level for 1011 *S. cerevisiae* strains, was downloaded from the “The 1002 Yeast Genome project” website (http://1002genomes.u-strasbg.fr/files/). For each QTL site identified in this study, the occurrence of the RM11-1a and YJM975α alleles was counted across strains in terms of number of strains (genotypes) carrying at least 1 copy of the allele, and of number of alleles present in each lineage over the total number of alleles in the called genotypes within the lineage (Info field = AN, VCFv4.1). Sites for which the reference or alternative alleles did not match the RM11-1a and YJM975α alleles from [34], were excluded from all analyses. Lineage assignment was based on [37] and mosaic lineages were excluded prior calculation of the phylogenetic distribution of the identified QTLs. SnpEff (v4.3) [80] was used to annotate and predict the effect of the variants.

### Bioinformatic analysis

Gene networks representing the most depleted and enriched deletion strains were made in STRING version 11.0 [81] and visualized using Cytoscape version 3.7.1 [82].

## Supplementary Information

**Additional file 1.** **Figure S1**. The absence or presence of *LEU2* influences the progeny’s phenotypes. Segregants with or without *LEU2* are labeled in yellow or grey respectively. **Figure S2.** Fraction of overall QTLs and the top 10 percent QTLs with strongest average effect on the trait compared to the distribution of all variants between the parental strains. **Figure S3**. Residual sucrose at the end of fermentation (16^o^P). N = 3 biological replicates. *P*-values are indicated by asterisk symbols (*: *p* <  = 0.05, **: *p* <  = 0.01, ***: *p* <  = 0.001, ****: *p* <  = 0.0001). **Figure S4**. Effect of swapping predicted QTL alleles on various phenotypes between the haploid strains RM11-1a (RM) and YJM975α (YJM). **A)** frameshift variant (394∆A^fs^) in *SUC2* and **B)** 184A > C in *ALD6*. Each point is represented as normalized mean ± STD of at least three biological replicates. *P*-values are indicated by asterisk symbols (*: *p* <  = 0.05, **: *p* <  = 0.01, ***: *p* <  = 0.001, ****: *p* <  = 0.0001). **Figure S5**. Swapping of variants that are linked to *IMA1* between the haploid strains RM11-1a (RM) and YJM975α (YJM) did not result in significant change of the traits where *IMA1* was mapped to. Each point is represented as normalized mean ± STD of at least three biological replicates. *P*-values are indicated by asterisk symbols (*: *p* <  = 0.05, **: *p* <  = 0.01, ***: *p* <  = 0.001, ****: *p* <  = 0.0001). **Figure S6**. Correlation between the *IMA1*-*MAL* genotype on chromosome VII and growth on maltose as the carbon source. **A)** Average sequencing coverage of the F_6_ segregants at the *IMA1*-*MAL* locus on Chromosome VII. **B)** Relative growth on maltose of segregants according to their parental genotypes at the *IMA1*-*MAL* locus. **Figure S7.** Identification of the causal variant underlying differences in isobutanol production **A)** Isobutanol production of the variants swapped between the parent strains RM11-1a and YJM975α. B**)** Isobutanol production of *URK1* knockout (KO) and WT strains of *S. boulardii*, Ethanol Red, and CEN.PK. Error bars represent standard deviations from three biological replicates. *P*-values are indicated by asterisk symbols (*: *p* <  = 0.05, **: *p* <  = 0.01, ***: *p* <  = 0.001, ****: *p* <  = 0.0001). **Figure S8**. Zoom-In subgraph of Fig. 1 (1/2). **Figure S9**. Zoom-In subgraph of Fig. 1 (2/2). **Figure S10**. Zoom-In subgraph of Fig. 2 (1/3). **Figure S11**. Zoom-In subgraph of Fig. 2 (2/3). **Figure S12**. Zoom-In subgraph of Fig. 2 (3/3).

**Additional file 2. Table S1**. Mapping analysis of the 18 different yeast traits relevant to industrial fermentations and biotechnological processes of all 1,125 previously sequenced inbred segregants (F_6_). **Table S2**. Mapping analysis of the 18 industrially relevant phenotypes of the prototrophic F_6_ segregants. **Table S3**. Overview of the identified QTLs, including the analysis of their distribution across natural *S*. *cerevisiae* strains (The 1002 Yeast Genome project). **Table S4**. The yeast strains used and constructed in the current study. **Table S5**. The oligonucleotides used in the current study.

**Additional file 3.** Phenotypic measurements of the industrially relevant traits from the 1,125 F_6_ segregants.

**Additional file 4.** Data used for plotting the main figures.

**Additional file 5.** Data used for plotting the supplementary figures.

## Data Availability

Raw whole-genome sequencing reads of F_6_ segregants and the 2 parental strains are available at https://www.ncbi.nlm.nih.gov/sra (SRR5634347–SRR5634826, SRR5629781–SRR5630260, SRR5630261–SRR5630452); genotypic data of the panel were obtained from She & Jarosz (2018). Phenotypic data of 1125 F_6_ segregants for the mapping experiments are included in the Additional file 3. Phenotypic data of the main and the supplementary figures are available in the Additional file 4 and 5, respectively. All custom codes and a detailed documentation of the mapping of the *IMA1-MAL* locus are freely available on GitHub (https://github.com/peaceway33/PWH-FermentationP2G). Correspondence and requests for materials and data should be addressed to Kevin Verstrepen. (kevin.verstrepen@kuleuven.be).
