# Supplementary material for: Massive QTL analysis identifies pleiotropic genetic determinants for stress resistance, aroma formation, and ethanol, glycerol and isobutanol production in Saccharomyces cerevisiae
Source: Biotechnol Biofuels. 2021 Nov 2;14:211. doi: 10.1186/s13068-021-02059-w (PMC8564995; doi:10.1186/s13068-021-02059-w)
Supplement: Supplementary file 1 — Additional file 1. Figure S1. The absence or presence of LEU2 influences the progeny’s phenotypes. Segregants with or without LEU2 are labeled in yellow or grey respectively. Figure S2. Fraction of overall QTLs and the top 10 percent QTLs with strongest average effect on the trait compared to the distribution of all variants between the parental strains. Figure S3. Residual sucrose at the end of fermentation (16oP). N = 3 biological replicates. P-values are indicated by asterisk symbols (*: p < = 0.05, **: p < = 0.01, ***: p < = 0.001, ****: p < = 0.0001). Figure S4. Effect of swapping predicted QTL alleles on various phenotypes between the haploid strains RM11-1a (RM) and YJM975α (YJM). A) frameshift variant (394∆Afs) in SUC2 and B) 184A > C in ALD6. Each point is represented as normalized mean ± STD of at least three biological replicates. P-values are indicated by asterisk symbols (*: p < = 0.05, **: p < = 0.01, ***: p < = 0.001, ****: p < = 0.0001). Figure S5. Swapping of variants that are linked to IMA1 between the haploid strains RM11-1a (RM) and YJM975α (YJM) did not result in significant change of the traits where IMA1 was mapped to. Each point is represented as normalized mean ± STD of at least three biological replicates. P-values are indicated by asterisk symbols (*: p < = 0.05, **: p < = 0.01, ***: p < = 0.001, ****: p < = 0.0001). Figure S6. Correlation between the IMA1-MAL genotype on chromosome VII and growth on maltose as the carbon source. A) Average sequencing coverage of the F6 segregants at the IMA1-MAL locus on Chromosome VII. B) Relative growth on maltose of segregants according to their parental genotypes at the IMA1-MAL locus. Figure S7. Identification of the causal variant underlying differences in isobutanol production A) Isobutanol production of the variants swapped between the parent strains RM11-1a and YJM975α. B) Isobutanol production of URK1 knockout (KO) and WT strains of S. boulardii, Ethanol Red, and CEN.PK. Error bars represent sta [file 13068_2021_2059_MOESM1_ESM.docx]

**Additional file figures**

**
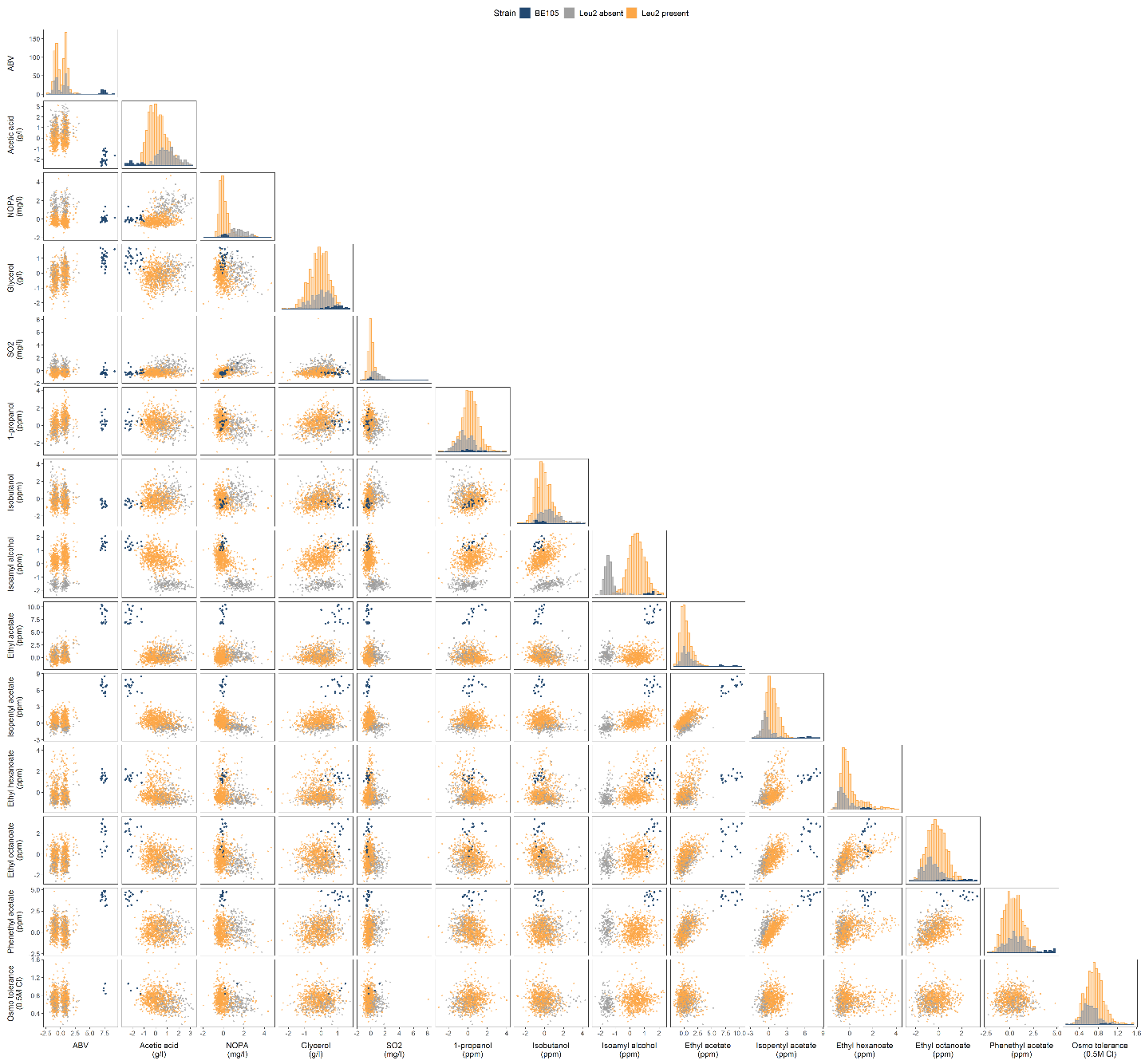
**

**Figure S1**. The absence or presence of *LEU2* influences the progeny’s phenotypes. Segregants with or without *LEU2* are labeled in yellow or grey respectively.


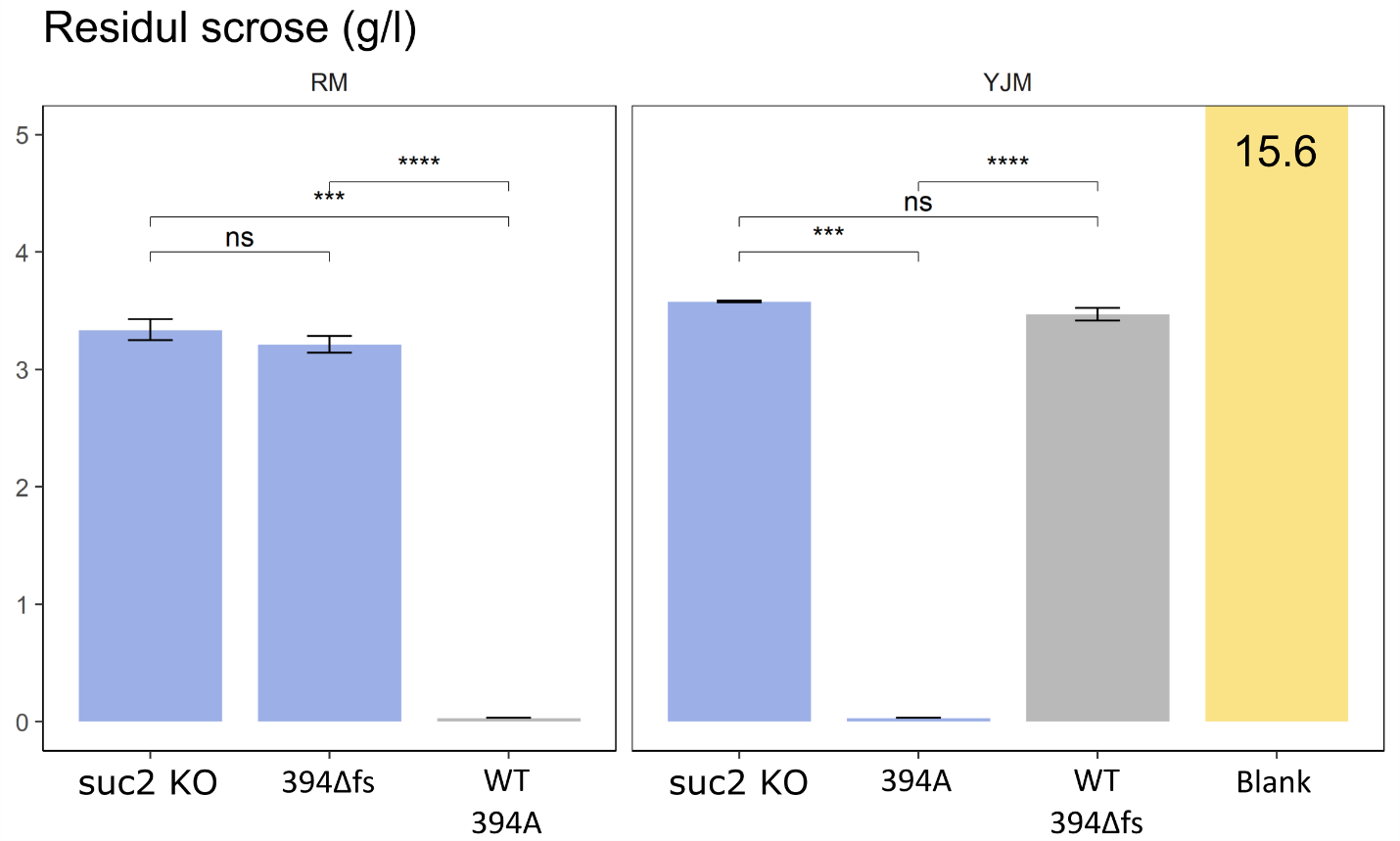


**Figure S2**. Residual sucrose at the end of fermentation (16^o^P). N= 3 biological replicates. P values are indicated with the level of significance (*: p <= 0.05 , **: p <= 0.01, ***: p <= 0.001, ****: p <= 0.0001).


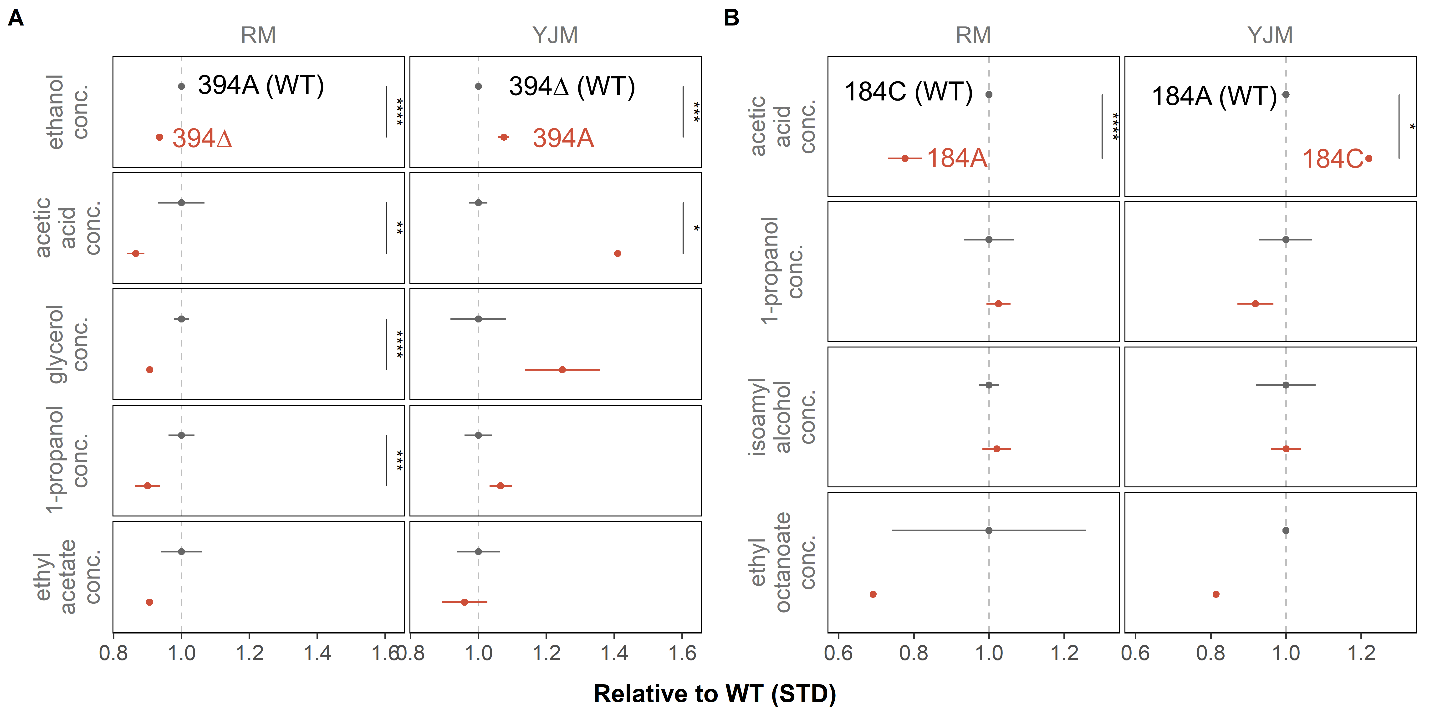


**Figure S3**. Effect of swapping the **A)** frameshift variant (394∆A^fs^) in *SUC2* and **B)** 184A>C in *ALD6* in the haploid strains RM11-1a (RM) and YJM975α (YJM) on the mapped traits (Table 1). Each point is represented as normalized mean ± std of at least three biological replicates. P values are indicated with the level of significance (*: p <= 0.05 , **: p <= 0.01, ***: p <= 0.001, ****: p <= 0.0001).


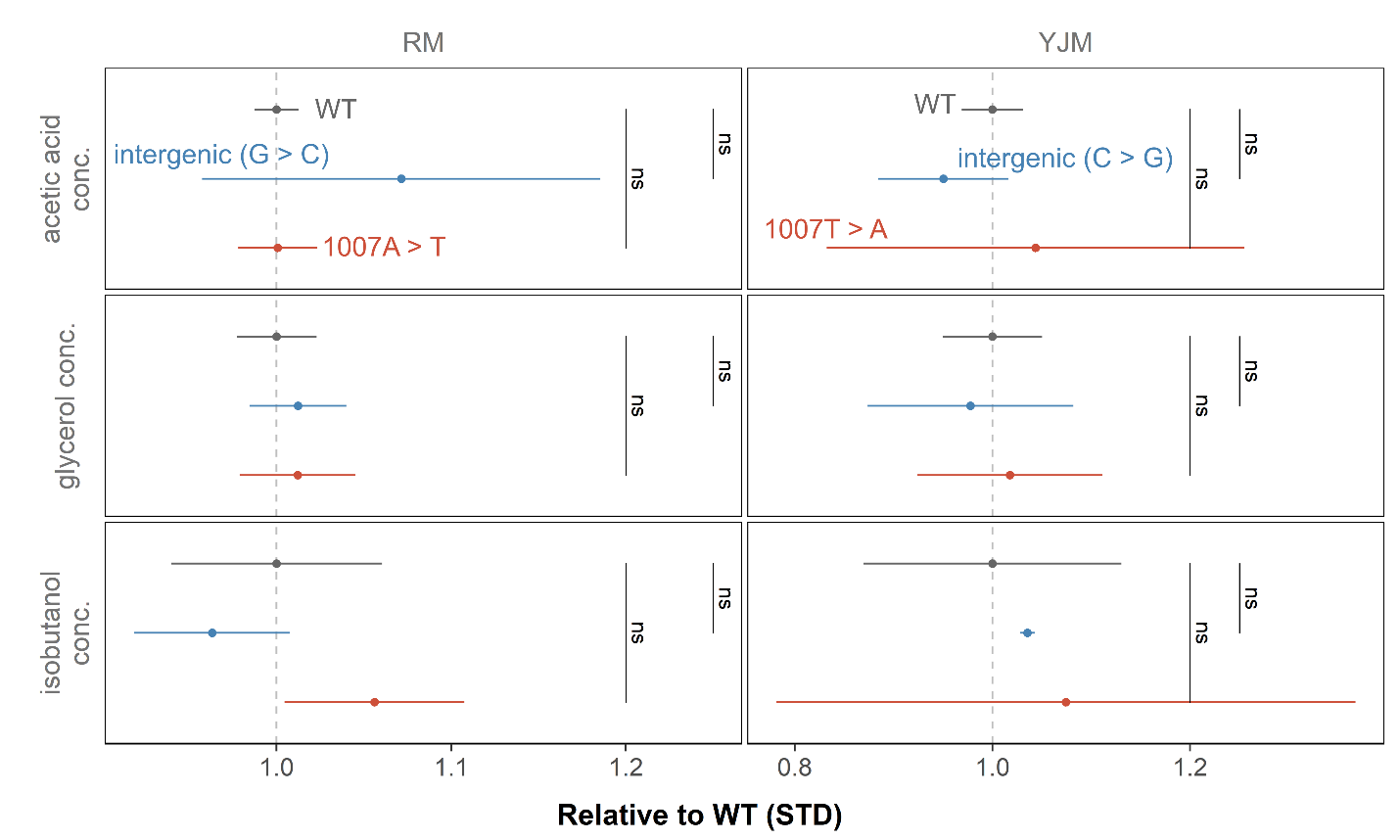


**Figure S4**. Swapping of variants that are linked to *IMA1* between the haploid strains RM11-1a (RM) and YJM975α (YJM) did not result in significant change of the traits where IMA1 was mapped to. Each point is represented as normalized mean ± std of at least three biological replicates. P values are indicated with the level of significance (*: p <= 0.05 , **: p <= 0.01, ***: p <= 0.001, ****: p <= 0.0001).


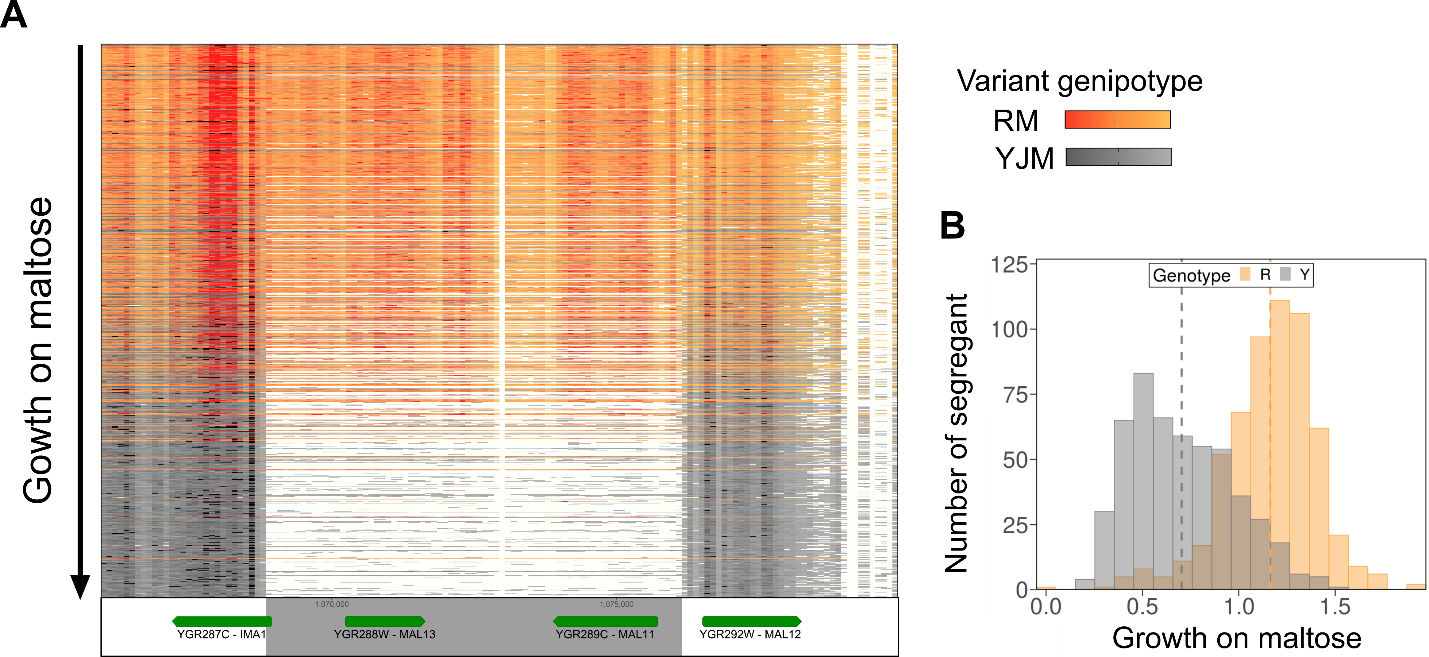


**Figure S5**. Correlation between the IMA1-MAL genotype on chromosome VII and growth on maltose as the carbon source. **A)** Average sequencing coverage of the F_6_ segregants at the IMA1-MAL locus on chromosome VII. **B)** Relative growth on maltose of segregants according to their parental genotypes at the IMA1-MAL locus.


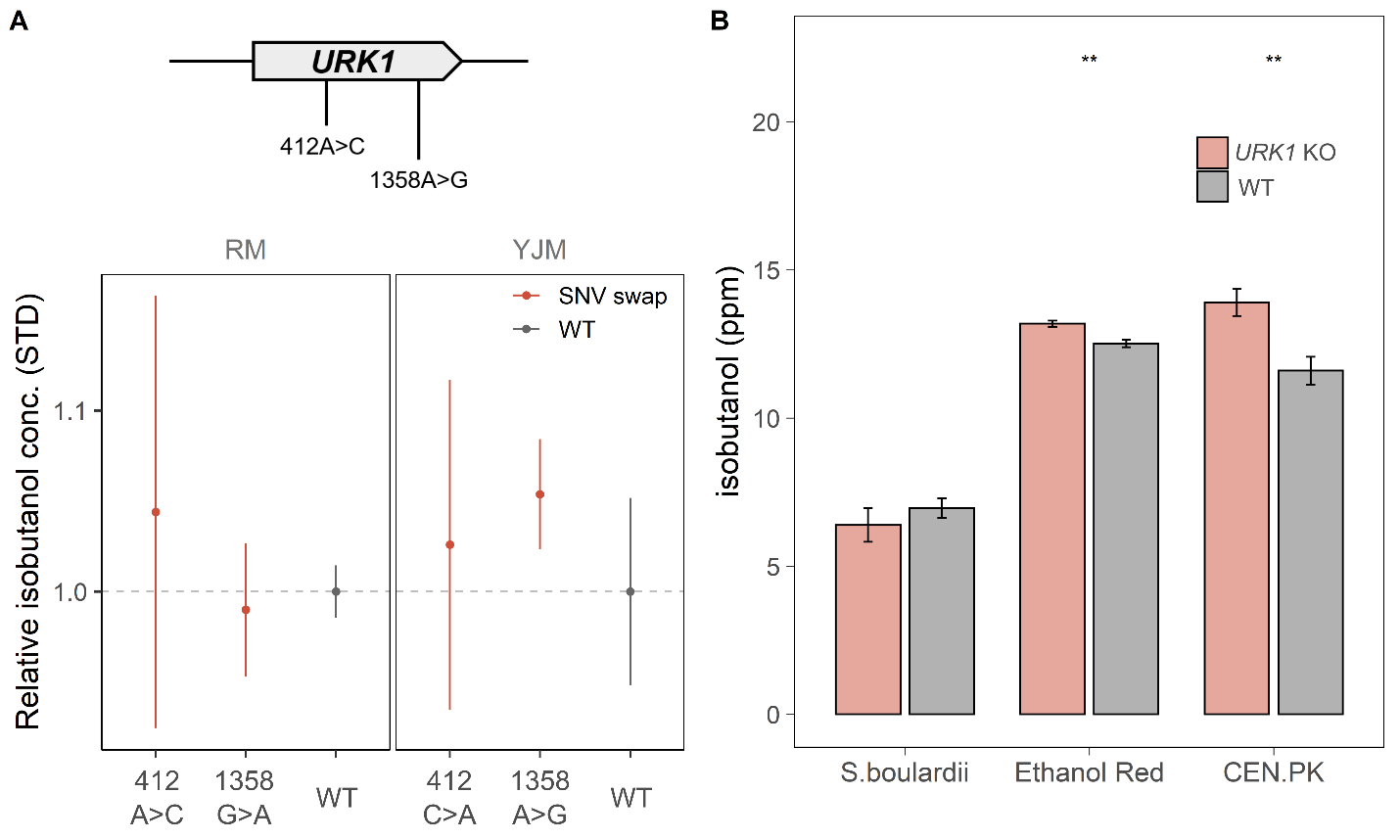


**Figure S6.** Identification of the causal variant of isobutanol production in the *URK1* locus. **A)** Isobutanol production of the variants swapped in the parent strain RM11-a and YJM975α. B**)** Isobutanol production of *URK1* knockout (KO) and WT strains of S. boulardii, Ethanol Red, and CEN.PK. Error bars represent standard deviations from three biological replicates. P-values are indicated with the level of significance (*: p <= 0.05 , **: p <= 0.01, ***: p <= 0.001, ****: p <= 0.0001).


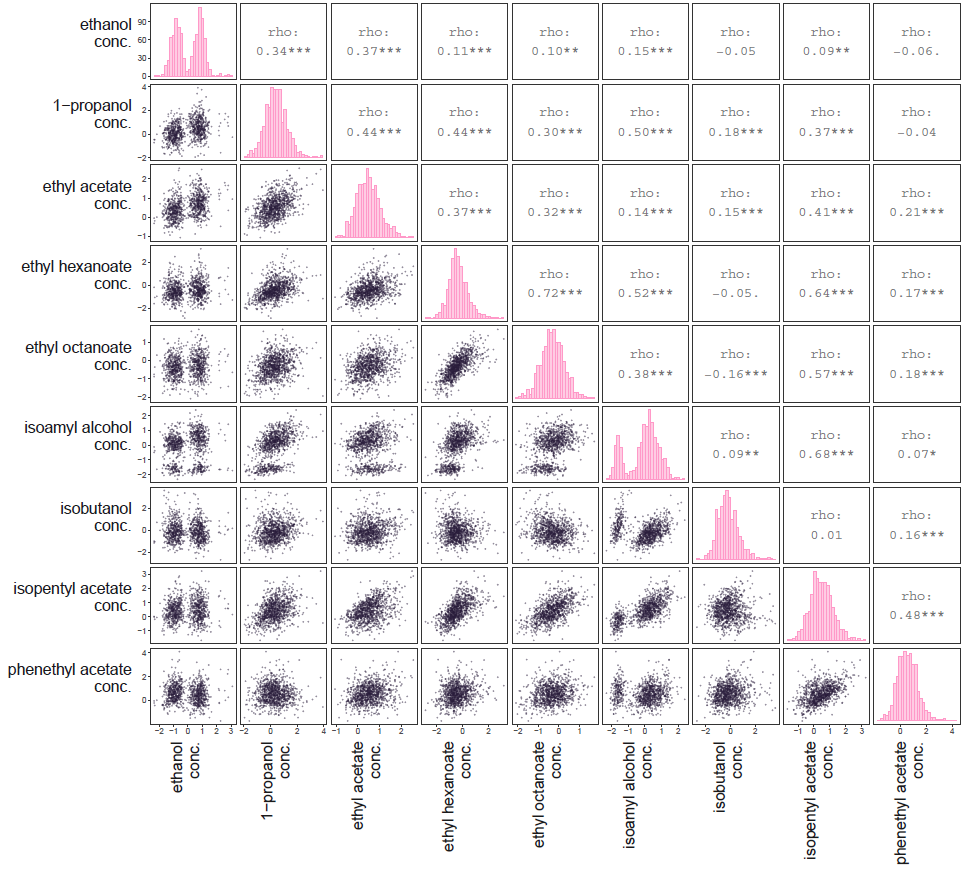


**Figure S8**. Zoom-In subgraph of Figure 1 (1/2).


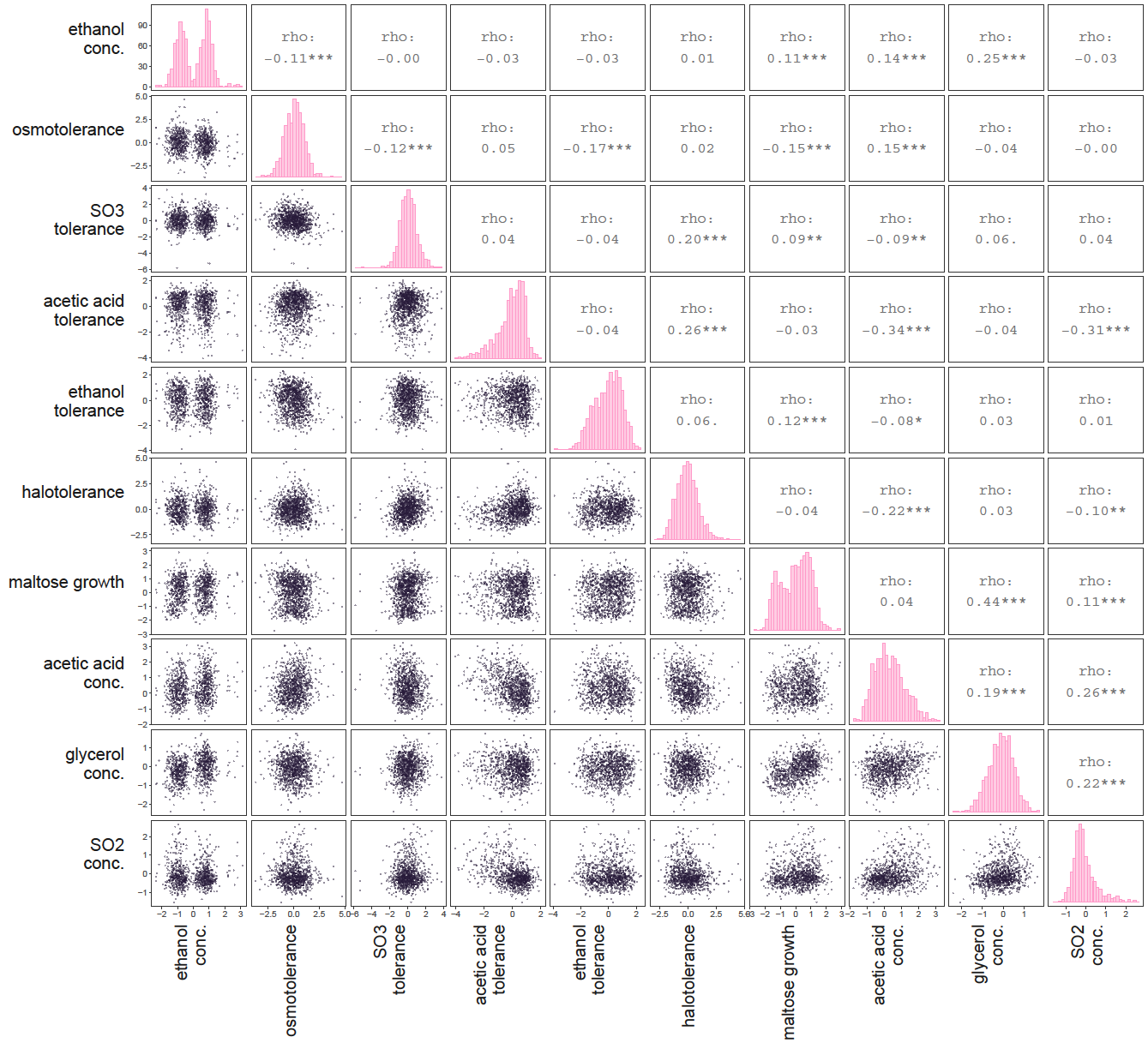


**Figure S9**. Zoom-In subgraph of Figure 1 (2/2).


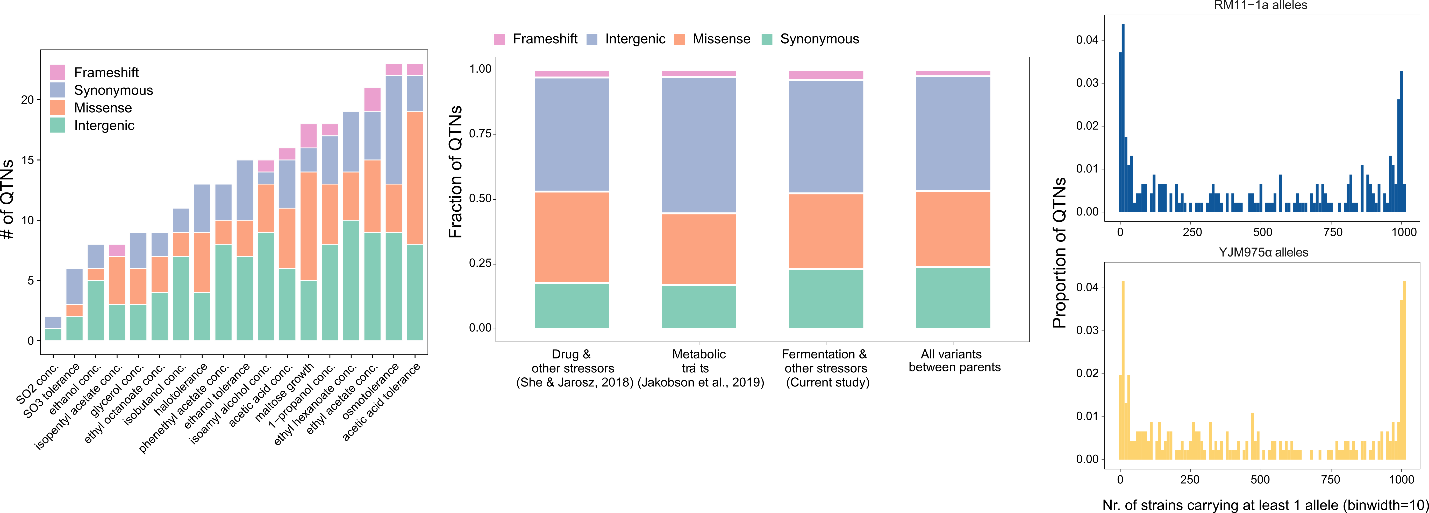


**Figure S10**. Zoom-In subgraph of Figure 2 (1/3).


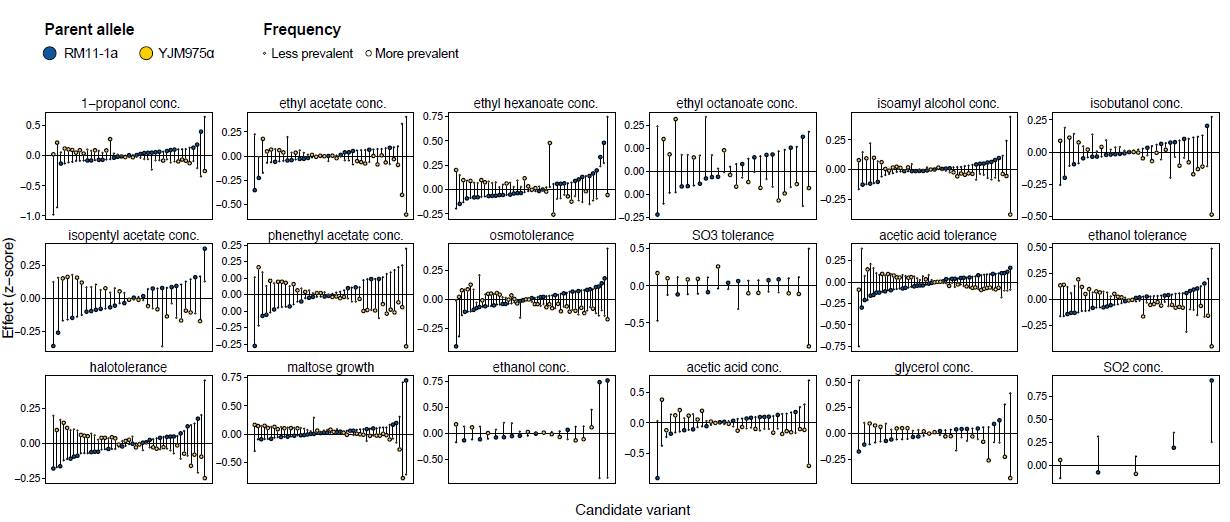


**Figure S11**. Zoom-In subgraph of Figure 2 (2/3).


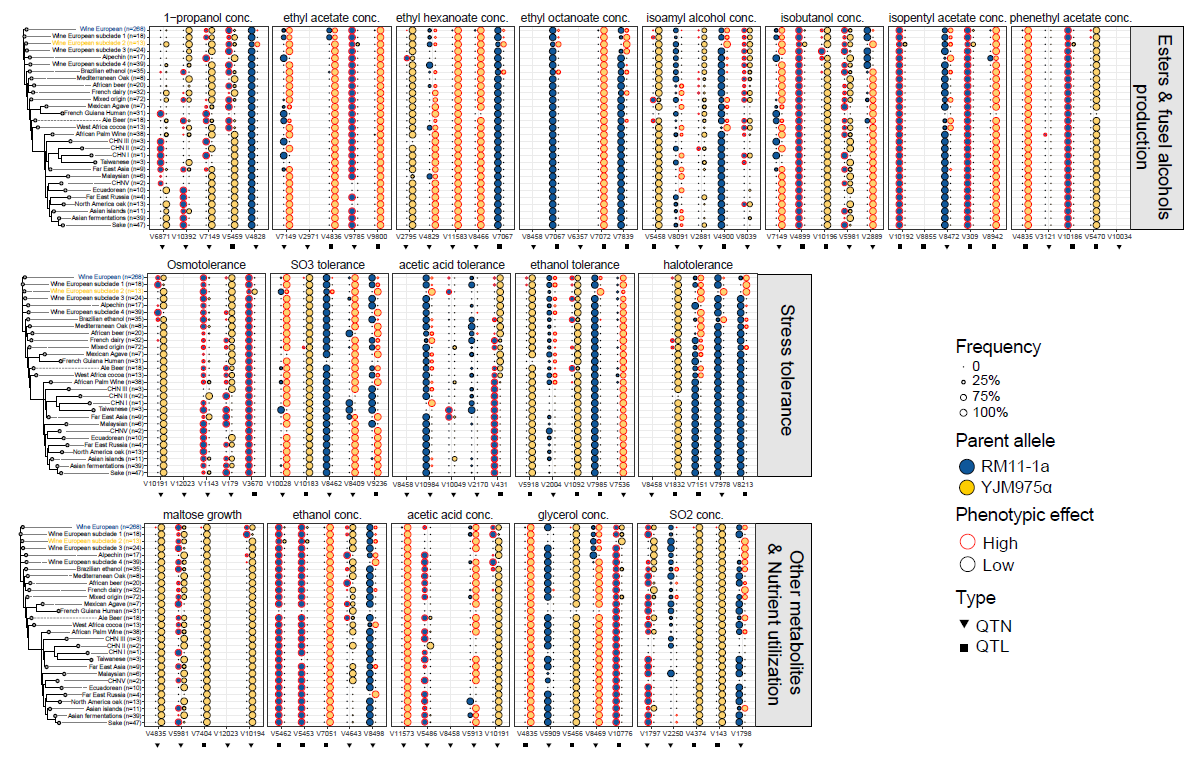


**Figure S12**. Zoom-In subgraph of Figure 2 (3/3).
